# Three-Dimensional RNA Structure of the Major HIV-1 Packaging Signal Region

**DOI:** 10.1016/j.str.2013.04.008

**Published:** 2013-06-04

**Authors:** James D. Stephenson, Haitao Li, Julia C. Kenyon, Martyn Symmons, Dave Klenerman, Andrew M.L. Lever

**Affiliations:** 1 Department of Medicine, University of Cambridge, Cambridge CB2 0QQ, UK; 2 Department of Chemistry, University of Cambridge, Cambridge CB2 1EW, UK; 3 Department of Pathology, University of Cambridge, Cambridge CB2 1QP, UK

## Abstract

HIV-1 genomic RNA has a noncoding 5′ region containing sequential conserved structural motifs that control many parts of the life cycle. Very limited data exist on their three-dimensional (3D) conformation and, hence, how they work structurally. To assemble a working model, we experimentally reassessed secondary structure elements of a 240-nt region and used single-molecule distances, derived from fluorescence resonance energy transfer, between defined locations in these elements as restraints to drive folding of the secondary structure into a 3D model with an estimated resolution below 10 Å. The folded 3D model satisfying the data is consensual with short nuclear-magnetic-resonance-solved regions and reveals previously unpredicted motifs, offering insight into earlier functional assays. It is a 3D representation of this entire region, with implications for RNA dimerization and protein binding during regulatory steps. The structural information of this highly conserved region of the virus has the potential to reveal promising therapeutic targets.

## Highlights

• The 2D structure of the HIV-1 5′ UTR RNA has been elucidated in a monomerized form
• The low-resolution 3D structure has been determined by FRET and simulated annealing
• Modeling has revealed an unpredicted kink turn

## Graphical Abstract

**Figure:**
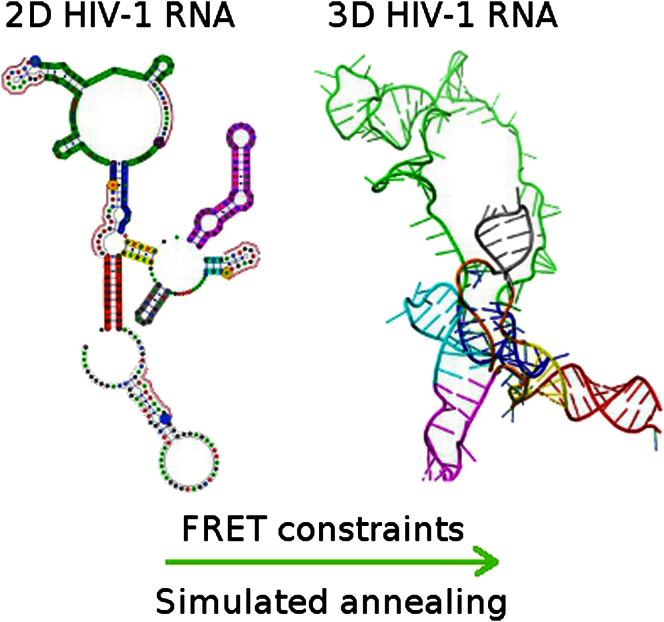

## Teaser

The highly conserved packaging signal region of HIV-1 RNA represents a potential therapeutic target. Stephenson et al. present a monomerized 2D structure of the region and a 3D model elucidated by FRET and simulated annealing which reveals an unpredicted kink turn.

## Introduction

Effective treatments now exist to suppress HIV replication, but high sequence variability and mutational escape contribute to the lack of an effective vaccine. Regions of high RNA sequence conservation provide attractive therapeutic targets. One such sequence is the 5′ untranslated region (UTR), present in all genomic HIV transcripts whose stringent conservation is attributable to the presence of many regulatory regions controlling reverse transcription (Aiyar et al., 1992), transcription (Aboul-ela et al., 1995), dimerization (Laughrea and Jetté, 1994) (necessary for packaging; Russell et al., 2004), and splicing (Harrison and Lever, 1992). These functions depend on recognition of structured regions of the RNA by viral and cellular proteins, exemplified by the viral Gag protein specifically binding the packaging signal, which allows the full-length viral genome to be distinguished from cellular RNAs and selectively encapsidated (Lever et al., 1989). Other viral and cellular protein interactions also occur here, but the structural basis for these is largely obscure due to the very limited available data on the three-dimensional (3D) conformation the RNA adopts. Small nucleotide perturbations of the sequence can cause catastrophic effects on viral infectivity (Harrison et al., 1998a), probably through effects on the global 5′ RNA structure during folding.

Several methodologies have been used to elucidate the secondary structure of the HIV-1 5′ UTR (Harrison and Lever, 1992; Baudin et al., 1993; Berkhout 1996; Harrison et al., 1998b; Berkhout et al., 2002; Paillart et al., 2002, 2004; Clever et al., 2002; Damgaard et al., 2004; Lu et al., 2011a), including, most recently, high-throughput selective 2′ OH acylation analyzed by primer extension (SHAPE) technology (Watts et al., 2009). These have proven useful in attributing functions to RNA regions. Most studies, however, consider mixed populations of monomeric and dimeric RNA. HIV-1 readily dimerizes via a kissing loop interaction between the palindromic sequence at the apical loop of stem loop 1 (Berkhout and van Wamel, 1996). The presence of dimers leads to potential confusion when assigning paired/unpaired states to nucleotides using biochemical data, since paired-like signals could be a result of local helices, long-range loop-loop intramolecular interactions, or intermolecular interactions between the monomeric components of a dimer.

The 3D structures of several small isolated fragments of the 5′ UTR have been elucidated by nuclear magnetic resonance (NMR) (Amarasinghe et al., 2000; Zeffman et al., 2000; Greatorex et al., 2002; Pappalardo et al., 1998; Lu et al., 2011b), but it is unknown how these structures relate to each other topologically since there are no published 3D solutions of large segments of HIV RNA. Traditional 3D modeling techniques such as X-ray crystallography and cryo-electron microscopy are not well suited to elucidating large RNA structures, due to the complexity and flexibility of the molecule (Shapiro et al., 2007). Such techniques are also rarely conducted in physiological conditions and cannot dynamically resolve structural changes (Simon and Gehrke, 2009) that may occur when proteins bind (Williamson, 2000). The difficulty in resolving the 3D structure of the 5′ RNA region in HIV-1 is also exacerbated by dimerization.

In order to avoid the confusing signals caused by mixed inter- and intramolecular RNA interactions in the dimer, we competed out dimeric RNA to produce a homogeneous monomeric species and, in close to intracellular conditions, solved the secondary structure of the major packaging signal region of HIV-1. We then used through-space distances derived from single-molecule fluorescence resonance energy transfer (SmFRET) experiments to build a 3D structure by molecular modeling. The resulting structure fits well with the known functions of this region and also with known short NMR-derived substructures. Apart from revealing the relative orientations and flexibilities of previously documented helix loop motifs, modeling reproducibly demonstrates an unpredicted kink-turn motif at the core of the structure, which is suggestive of a protein binding site and which explains the effects on genome packaging of previous mutations. This demonstration of this methodology to generate a large viral RNA structure in three dimensions indicates the power and versatility of the technique. The information on flexibility and general helix position of the HIV-1 5′ UTR RNA presented here has the potential to facilitate structural analysis of protein binding and to aid intelligent drug design.

## Results

### Monomeric Two-Dimensional Prediction

**Figure 1:**
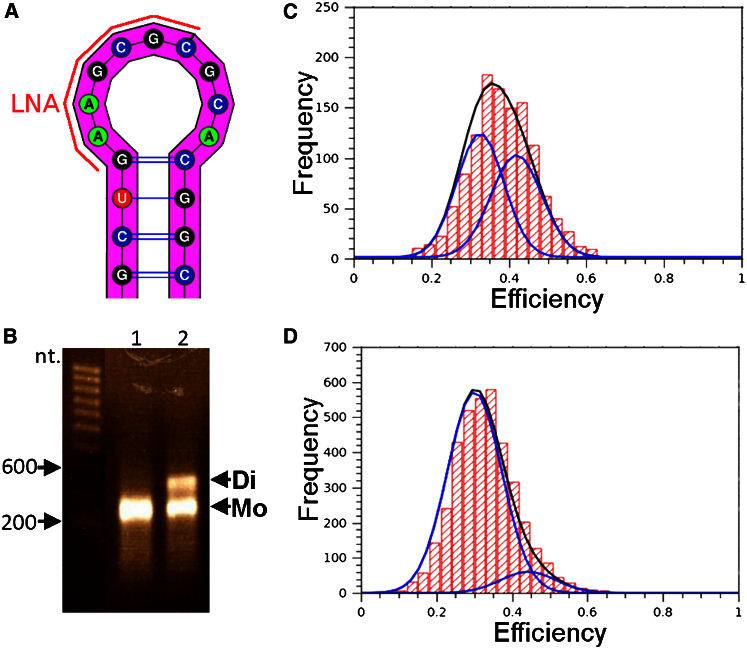
The Effect of Adding LNA Directed to the Dimerization Signal of HIV-1 5′ UTR RNA (A) LNA recognition site at the dimerization palindrome located at the loop of SL1. (B) Native agarose gel showing 310 nt HIV-1 5′ UTR with (lane 1) and without (lane 2) LNA, monomer (Mo) and dimer (Di) labeled. (C and D) FRET-derived histogram with Gaussian curves overlaid from labeled RNA (C) without LNA and (D) with LNA. See also Figure S3.

HIV-1 RNA dimerization is well studied (Berkhout and van Wamel, 1996), and the intermolecular interaction at positions 257G–262C (Figure 1A) has been elucidated to fine detail (Cao and Chen, 2011). By electrophoresis of nucleotides (nt) 104–413 of the HIV-1 packaging signal RNA, we have demonstrated that dimerization also occurs for our subsection length under the conditions used in our FRET experiments (Figure 1B, lane 2). In order to avoid confusing inter- and intramolecular interactions in either two-dimensional (2D) or 3D modeling, we resolved the 310 nt packaging signal (*psi*) region of HIV-1 into a pure monomeric species using a blocking locked nucleic acid (LNA) (5′ GCGCUUC 3′), complementary to 4 of the 6 nt (Figure 1A) involved in dimerization initiation (an LNA that annealed to all six would itself dimerize). Successful monomerization was shown by the reduction from two bands at ∼600 nt and ∼300 nt to one band at ∼300 nt by electrophoresis (Figure 1B). This was further confirmed by the elimination of the intermolecular FRET efficiency peak as shown in Figures 1C and 1D.

**Figure 2:**
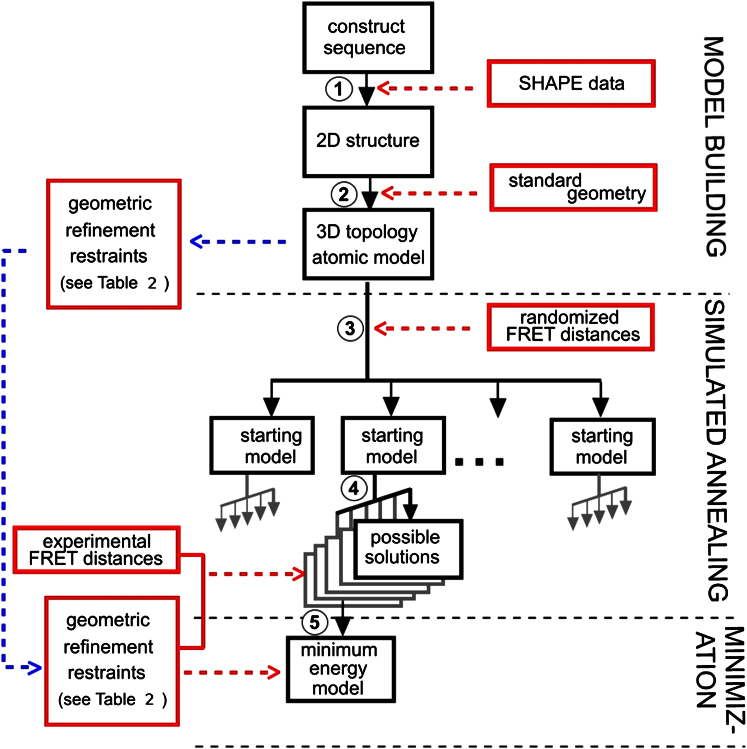
Flow Diagram of the Model Building Steps to Build a 3D Structure from Primary Sequence Black boxes indicate RNA models, red boxes indicate restraints for model building, red dashed arrows indicate data input, and blue dashed arrows indicate data output. Model building steps 1–5 are discussed in the Results and Experimental Procedures.

Constructing a 3D model from an RNA sequence can be considered a five-step process as shown in Figure 2. The first of these steps requires a prediction of the 2D organization of the sequence. This we achieved using SHAPE technology (Wilkinson et al., 2008), which relies on the fact that conformationally flexible nucleotides are preferentially reactive to N-methylisatoic anhydride (NMIA). Using SHAPE, we resolved unpaired from paired nucleotides in the monomeric 5′ HIV-1 RNA by considering their chemical reactivities (Table S1 available online). Five replicates were performed, and sites containing inconsistent biochemical information were not used in the modeling. SHAPE reactivity values were entered as pseudo-free-energy restraints into the RNA secondary structure prediction software RNAstructure (Reuter and Mathews, 2010). The reactivities served to represent base pairing probabilities. Such “soft” restraints have an advantage over “hard” restraints (which force states) as they allow greater flexibility in the model, thus permitting a more rigorous exploration of structure space. By accepting only unpaired constraints, we minimized any risk of false designation of base pairing caused by long-range loop-loop interactions.

**Figure 3:**
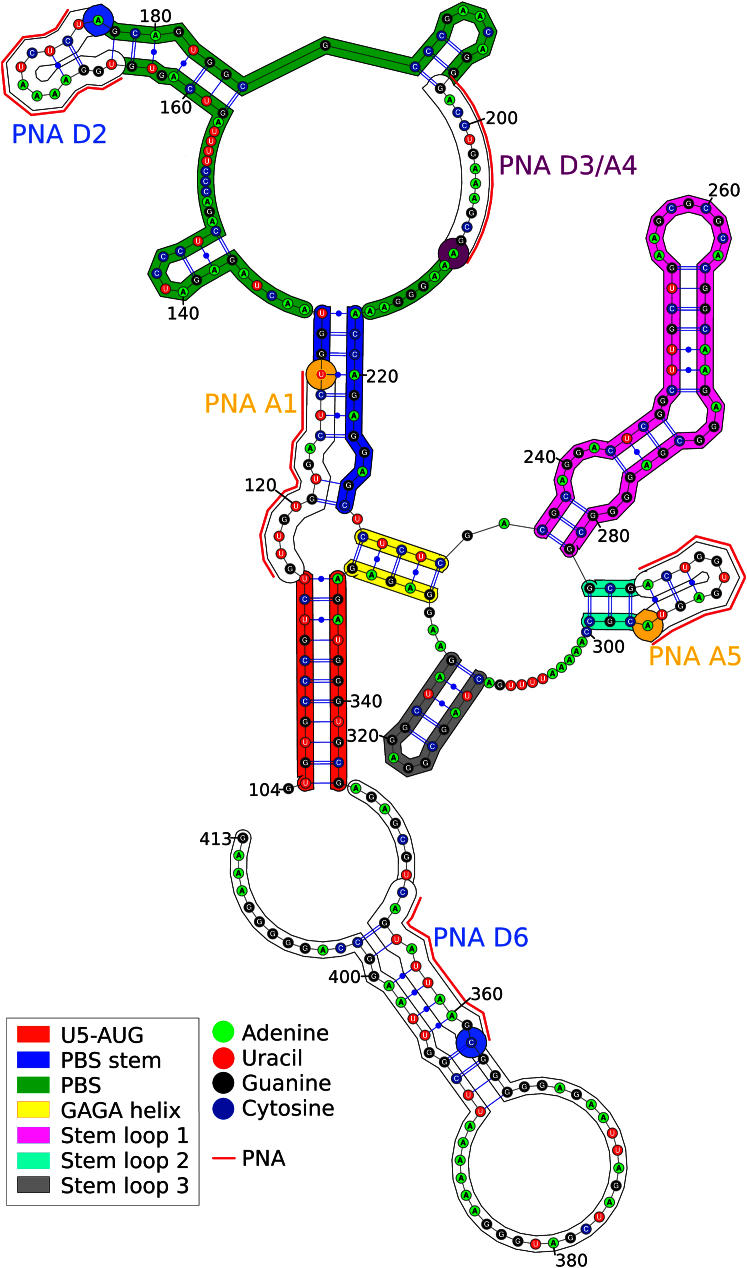
2D Model of the HIV-1 5′ UTR RNA based on SHAPE Data Collected from a Monomerized RNA Population The fluorophore locations are displayed directly over the nearest nucleotide to the linker as the model distances are considered to be between the C1′ atom of these bases during computational modeling. PNA binding locations are shown as red lines (PNA sequences can be found in the Experimental Procedures). Regions are colored to allow comparison with the 3D model in Figure 5. The 3′ end extending beyond U5-AUG (345–413) permitted SHAPE (Table S1) and RNase probing (Figure S1) to the terminus of the U5-AUG helix and provided a fluorophore binding site. See also Figure S1 and Table S1.

The SHAPE reactivities that we derived and on which the modeling was based differed in part from previous SHAPE analyses of the region (Wilkinson et al., 2008; Watts et al., 2009). This is not surprising, as the RNA we used was of a different length from that used by Watts et al. (2009) or Wilkinson et al. (2008) and SHAPE inconstancies have previously been recorded between different sized RNAs even in the same lab (Stephenson and Lever, 2009). Additionally, we probed a strictly defined single species of monomerized RNA to avoid any potential confounding issues of intermolecular interactions affecting the reactivities. We further constrained our structure by widely accepted and well-documented intramolecular interactions (as discussed in Experimental Procedures) to arrive at the monomeric 2D RNA structure prediction shown in Figure 3. It is important to note that the 2D model we present (and, indeed, the 3D model) represents monomeric RNA of a particular length under specific conditions of buffer and temperature.

A second independent biochemical technique, ribonuclease (RNase) digestion and reverse transcriptase pausing (Harrison and Lever, 1992), was performed on the monomeric RNA. In this technique, different RNases preferentially cleave different nucleotides in a structure- and/or sequence-specific manner. This causes dissociation of reverse transcriptase at that nucleotide and the production of a defined length cDNA detectable by electrophoresis. Cleavages were mapped on to the predicted secondary structure and further validated the 2D structural prediction (Figures S1A–S1C). As with any biochemical technique, both SHAPE and reverse transcriptase pausing have inherent limitations, both experimentally and analytically, so the high degree of agreement adds weight to the model validation.

Although the 2D model is useful in indicating local Watson-Crick interactions, other interactions such as loop-loop interactions, base stacking, and sugar edge interactions mean that RNA structures are not well represented on a plane. Without biochemical data on the distances between RNA positions through space, these interactions cannot be modeled. However, previous knowledge regarding geometric constraints of bond angles and lengths, as well as the geometry of helices can be used to generate a crude 3D representation of the 2D model. We used the software RNA2D3D (Martinez et al., 2008) to convert our 2D representation of the RNA into an all-atom 3D version as a starting structure for 3D modeling (Figure S2A) (step 2 in Figure 2). This structure was constructed using standard RNA geometry but without any user-added constraints. It does not represent a prediction of the 3D shape of the RNA but simply shows a 3D representation of the 2D structure.

Starting the modeling process from our initial 3D representation may have limited the amount of searchable structure space; for example, a helix may never be able to orient itself successfully if sterically blocked by another part of the structure. In order to increase the proportion of structure space searched and thus to maximize the probability of achieving the ideal solution; random starting structures were constructed by applying sets of pseudoexperimental restraints using the Crystallography and NMR system (CNS) (Brunger, 2007), as explained in Figure S4A and represented by step 3 in Figure 2. These large displacements were accommodated within the overall structure by arbitrary backbone rotations of the single-stranded sections. During this process, all 2D predicted base pairings were maintained and helices were restricted to the basic A-form. The result was 10 very different 3D starting models (r0–r9), each with potentially different searchable structure space localities.

### FRET Analysis

In order to predict the most likely relative orientations of features in the RNA, distances were required between several points. FRET has been used previously for this purpose in solving the human telomerase core RNA structure (Gavory et al., 2006). The principle of FRET is that an excited fluorophore (known as the donor) preferentially transfers energy nonradiatively to a nearby acceptor fluorophore rather than itself fluorescing. If the emission wavelength overlaps the acceptor excitation wavelength, the result is a reduction in donor fluorescence and an increase in acceptor fluorescence. The magnitude of energy transferred indicates the distance between the fluorophores. Annealing fluorophore pairs to the RNA and measuring fluorescence intensity therefore yields information on the distances between the annealed points through space.

Fluorophores Atto488 (donor) and Atto647 (acceptor) were bound via linkers to 11–13 nt peptide nucleic acid (PNA) oligonucleotides, which in turn had sequences complementary to the RNA target regions. Fluorophore positions were chosen so that the pairs could not be separated by more than 10 nm (the longest useful FRET distance) and also to provide maximum coverage of the structure. The sites were also sequence unique from one another, had favorable binding energies, and caused minimal structure change as determined in silico (Figure S1D). The specificity of the PNAs and the absence of global structural changes on binding were confirmed by reverse transcriptase pausing. This demonstrated distinct single pauses for PNA binding locations but no other cleavage pattern changes between RNAs with and without annealed PNAs (Figure S1E). Further evidence of the specific binding of each PNA is apparent in Figure S3, which consistently shows either one or two distinct FRET efficiency peaks. If there were more binding sites, there would be additional distinct peaks; similarly loosely bound fluorophores would cause a continuous signal across all efficiencies.

Unlike in previous FRET RNA analyses, many of our PNAs were designed to anneal to base-paired regions of the RNA. This has generally been avoided in previous studies for fear of inducing a global structural change. Several reasons make this approach reasonable in our case. Most important is the fact that the PNAs are added after the RNA has already folded into a stable structure. It has been shown previously that PNA oligonucleotides can displace an RNA helix (Peffer et al., 1993) due to the higher affinity of PNAs for RNA than RNA has for itself (Uhlmann et al., 1998; Natsume et al., 2007). Adding the PNA before RNA folding would likely change the folding hierarchy and cause a global structural change. It is, however, thermodynamically implausible that the already folded structure, which is stabilized by 64 canonical base pairs, would change on addition of a short oligonucleotide to one helix. Since it is prohibitively complex to predict how annealing PNAs will affect local and global structures, biochemical assessment is invaluable (Figure S1E), and our combined findings confirm that, while local structure is perturbed by PNAs, global structure is not.

SmFRET experiments were performed using all combinations of donor Atto488 (D2, D3, D6) and acceptor Atto647 (A1, A4, A5) pairs except for pair D3/A4, which anneal to the same sequence (Figure 3). The low RNA concentration meant that each photon burst captured was the result of a single fluorophore emission. Gaussian curves were fitted to frequency/efficiency FRET histograms (blue lines in Figure S3), which clearly showed two profiles for each pair. These were interpreted as the intramolecular FRET efficiency within a monomer and the intermolecular FRET efficiency between RNA in a dimer, as the latter was reduced to near-negligible levels on addition of LNA (Figure 1).

Apparent FRET efficiencies, *E*_app_, of each burst were calculated according to E_app_ = n_A_/(n_A_ + γn_D_), where n_A_ and n_D_ are the acceptor and donor counts, respectively.

γ = (ϕ_A_η_A_)/(ϕ_D_η_D_) is a factor accounting for the difference in the quantum yields, ϕ_A_ and ϕ_D_, and detection efficiencies, η_A_ and η_D_, for the acceptor and donor channels, respectively. This factor has been previously measured to be close to 1 for our set-up. Prior to calculating interfluorophore distances from FRET efficiencies, each repeat measurement was weighted by its signal:noise ratio in order to decrease the influence of more ambiguous measurements.

### Distance Calculations

The degree to which fluorophores are able to orient themselves favorably has an effect on the calculation of interdye distances from energy transfer efficiency. Since our dyes were tethered by a linker to each PNA, completely free rotation was unattainable; other dye interactions may also have played some role in constraining free rotation. Rotational freedom was therefore measured by experimental anisotropy experiments (Figure S2B), which uncovered the fact that neither the donor nor acceptor fluorophores rotate freely, probably due to some attraction to the RNA (this is the simplest but not the only possible explanation for the increased anisotropy). The orientation factor for the most extreme case (the donor) was corrected accordingly when calculating interfluorophore distances from FRET efficiencies.

The donor-to-acceptor separation distance, r, was calculated by the equation E = 1/[1+(r/R_0_)^6^], with R_0_ being the Förster distance (6.68 nm) of the Atto488 and Atto647 pairing as calculated from experimental anisotropy measurements (Figure S2B).

Not all Gaussian curves were the same width; a common contributor to peak width is the random fluctuation in fluorescence signal intensity in both channels called photon shot noise. Our FRET peaks were compared with a control which showed that shot noise could not entirely account for the peak widths (Supplemental Experimental Procedures). This indicates that there is some other fluctuation process contributing to the E(_app_) histograms. As the linker is identical in all cases, we suggest that the flexibility of the RNA linking the two dyes in the monomer may also contribute to widening of the histogram. We therefore used the area:height ratio of the histograms to calculate a distance tolerance for each pair used during the modeling where dynamic pairs were permitted a greater range of distances centered around their mean distance (Table S2).

The values used for restraining RNA 3D modeling were therefore the monomer distances, weighted to account for signal. The dynamic potential between the fluorophores (Distance Tolerance column in Table 1) was also used to define a range around the weighted distance where no energy penalty would be applied during modeling. These are marked as experimental FRET distances in step 4 of Figure 2.

**Table 1:** The Weighted Mean FRET Efficiencies of the Fitted Gaussian Curves Representing the Monomeric Species for Each Pair of Fluorophores

PNA Pair	Weighted FRET Efficiency	Distance (Å)	Histogram Area:Height Ratio	Distance Tolerance (Å)
A1 + D2	0.31	88.69	0.05	5.10
A1 + D3	0.50	65.39	0.04	1.90
A1 + D6	0.41	71.01	0.08	3.62
A4 + D2	0.41	69.64	0.05	2.42
A4 + D6	0.46	73.34	0.08	3.81
A5 + D2	0.44	70.98	0.05	2.34
A5 + D3	0.48	69.98	0.06	2.57
A5 + D6	0.47	62.44	0.03	1.43

The distance calculations from efficiency values (including experimentally derived anisotropy results) (Figure S2B). The area:height ratio of the monomeric curve (Table S2) is used to calculate a distance tolerance for each pair during modeling.

See also Figure S2B and Table S2.

FRET confers several advantages over other structural techniques, including the ability to consider flexibility and to alter conditions in real time. By using distance tolerance values during modeling, derived from efficiency curve widths, we were able to consider a range of distances rather than a static value, mirroring the degree of flexibility between fluorophore pairs. This allows a less prohibitive structure space search and more closely mimics the dynamic movement of RNA in solution.

### 3D Modeling

We used simulated annealing in the established CNS program to model our RNA as it provides a proven environment that is flexible enough to be extended beyond standard crystallographic or NMR refinements. Simulated annealing is a probabilistic metaheuristic that searches for a good approximation to a global optimum and is often used in structure modeling due to the large potential structure space. It searches conformational space by applying displacements to coordinates as if they are at high temperature.

During the search step, the FRET distances and SHAPE base pairing are expressed in CNS as distance restraints; that is, as elastic-like forces with an energy penalty set by a force constant. The search for a solution to the experimental distances is conducted as part of an energy minimization. Additional restraints, expressed as energy penalties to minimize final distortions, are also incorporated in the refinement (Table 2). These include energy penalties on unlikely bond lengths and angles, close contacts, and nonplanarity. Hydrogen bonding is known to be insufficient to produce regular double helical conformation, and so additional restraints are incorporated on torsional angles and distances between the phosphodiester backbones. One common distortion is the “laddering” of the base-pair regions where, perhaps owing to shearing forces from FRET restraints, the helical regions become overextended. We therefore introduced pseudobonds running along and across each double helical element, which act as crosslinking restraints to maintain the double helical conformation. They are established between the center of first and last base pairs in each double helical element and also diametrically across the helical axis from the backbone of one strand to the other. In this case, the restraints are offset in sequence owing to the fact that A-form base pairs are angled to the long helical axis.

**Table 2:** Restraints Used in Simulated Annealing

Type of Restraint	Details	Notes
Experimental FRET distances	Treated as NOE distances	Additional distance to deal with linker length
Experimentally determined secondary structure	Base pairing	H-bond distances
	Base pair planarity	Not constraining to allow for “propeller” twist of base pair
	Double-helical backbone torsional restraints	Based on values for A-type helix from XPLOR standard library
	Restraints on ribose ring to favor the 3′-endo pucker	Based on standard CNS values
Restraints to prevent “laddering”	Staggered strand-to-strand restraints across base pairs	Staggering is to allow for inclined angle of base pairs in A-form helix
(Applied only to helical regions)	End-to-end restraints on double helical sections	Length adjusted to account for differing numbers of base pairs
	Backbone torsional restraints	Based on X-PLOR values for nucleotides in A-form helix

See also red boxes in Figure 2. The final structure after simulated annealing is shown in Figure 5.

To improve the probability of finding the best solution, we sampled disparate parts of solution space by starting simulated annealing from different initial models (r0–r9).

Torsional simulated annealing was initiated in CNS from these 10 random starting models concurrently multiple times with random starting trajectories (five separate outputs were collected for each set of starting coordinates). The large differences in each starting structure allowed a sizable proportion of structure space to be searched, and the five initial trajectories from each increased the search space further. Conventionally, more starting structures are used (Larson et al., 2002), but they traditionally vary far less from each other and, therefore, sample local space more thoroughly but are less likely to sample very different structure space regions.

Following high-temperature simulated annealing, each structure was subjected to a further annealing at room temperature in order to investigate the local structure space more thoroughly (Minimized columns, Table S3). As a simplification to the model, the fluorophore atoms were not explicitly included in the simulated annealing runs. Instead, the distances used for modeling were between the C1′ atoms of the nucleotide closest to the fluorophore (Figure S2A). One key improvement for low-resolution data that is currently only available in the CNS system for protein refinements is the use of a knowledge-based probability potential for rotameric states. Rotameric behavior has been described for RNA (Murray et al., 2003), and including these as probabilistic restraints improves low-resolution RNA refinement.

The model fit for each FRET constrained structure was calculated by multiplying the root-mean-square deviation (RMSD) distance from the real FRET distances by the energy penalty remaining. The best representative structure from each of the 10 starting models is shown in the FRET Restrained columns in Table 3. The final stage for these 10 structures saw the removal of all FRET constraints and further room temperature simulated annealing. This step was introduced to establish that the conformation of the RNA was “native-like” and not artificially produced by the force constant of the FRET restraints (Relaxed columns in Table 3). The fit for the relaxed structures was judged in the same way as for the FRET constrained structures, and the overall model fit was the sum of these values for each structure (Model Fit column, Table 3).

**Table 3:** Ranked Model Fit Data from Different Starting Models

Starting Model	FRET Restrained		Relaxed		Model Fit
	Distance (Å)	Energy	Distance (Å)	Energy	
1	2.47	1,376.15	5.14	1,159.74	9,363.63
3	3.91	1,332.72	7.54	1,179.79	14,114.81
4	3.64	1,507.23	7.33	1,235.32	14,546.15
6	3.46	1,381.98	8.35	1,182.64	14,660.24
8	3.07	1,436.72	8.79	1,190.77	14,881.17
7	3.06	1,472.87	9.45	1,179.88	15,658.02
0	3.8	1,466.86	8.47	1,229.8	15,995.39
2	3.12	1,376.69	10.02	1,200.13	16,320.57
9	3.58	1,453.06	11.51	1,195.88	18,968.92
5	9.69	1,436.52	20.99	1,184.63	38,794.73

Distance measurements are the average distance differences of the model from the measured values. Energy is the energy penalty remaining after modeling. The FRET restrained columns show the model when it is heavily penalized for failing to satisfy the distance constraints, and the relaxed columns show the same data when the restrained models are unconstrained. Model fit is the sum of the distance × energy products for restrained and relaxed.

See also Table S3.

### Structure Refinement

**Figure 4:**
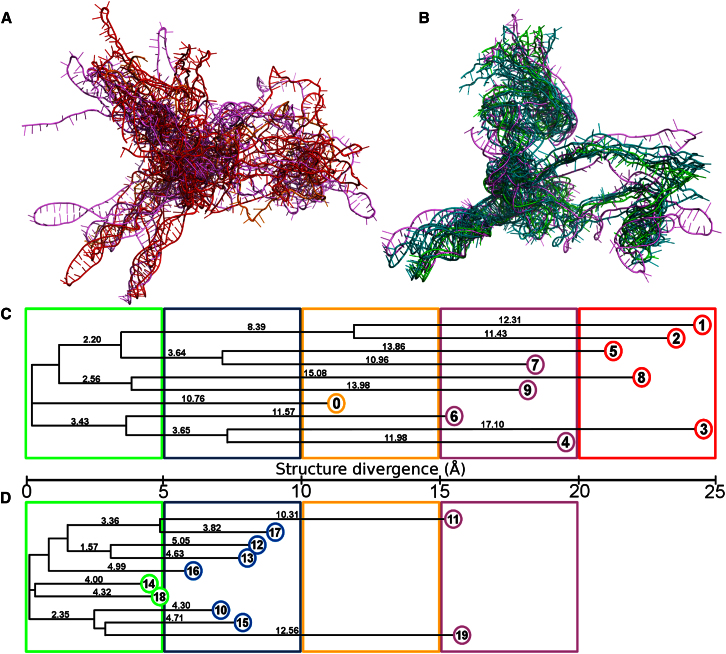
Comparison of the Structural Models Generated from Different Starting Models and from a Single One (A) The 10 structures generated from simulated annealing from different starting structures (r0–r9) colored according to the average structural divergence of that model from the other models. The colors are scaled from green, indicating very little structural variation, to red, indicating large structural variation. (B) The 10 structures all generated from the “1” starting structure (10–19) and colored as in (A). (C) Dendrogram constructed by neighbor joining from the pairwise structural variation matrix in Figure S4C from distances between structures in (A). Adding the branch scores along the path between two structures equates to the structural deviation between them in angstroms. Colored boxes show the minimum distance between structures, so that two red structures are at least 40 Å apart and two blue structures are at least 10 Å apart. The colors are the same as the structures in (A). (D) Dendrogram constructed by neighbor joining from the pairwise structural variation matrix in Figure S4D from distances between structures in (B). The scale and coloring scheme is the same as in (C) and the dendrogram shows that smaller average distances occur between models when starting from the same structure than between those when starting from different structures. See also Figure S4.

Simulated annealing does not guarantee finding the global optimum solution, as searching every possible structural conformation is currently computationally intractable. The final structures from the random start models may be considered local optima (some of which may have converged). Although we cannot know whether any of these is the global optimum, we make the assumption that the most likely candidate is the most energetically minimized local solution (structure 1 in Table 3). We then searched more thoroughly in the surrounding structure space of that model in order to optimize the solution. Different random trajectories were given to the starting structure r1 (Figure S4B), and the result of high and low temperature annealing was ten structures, termed 10–19 in Table 4. The overall pairwise RMSD was then calculated for structures 0–9 (different starting models, shown in Figure S4C) and also for structures 10–19 (same starting model, Figure S4D). A comparison of the neighbor joining built dendrograms from the similarity matrices revealed dramatically increased similarity among structures 10–19 (Figure 4), compared with 0–9, suggesting that the search had been refined.

**Table 4:** Ranked Model Fit Data from Structures from a Single Starting Model

Model	FRET Restrained		Relaxed		Model Fit
	Distance (Å)	Energy	Distance (Å)	Energy	
14	2.50	1,237.71	6.24	1,089.90	9,902.12
18	2.58	1,334.66	5.87	1,103.34	9,917.69
17	2.32	1,302.35	6.92	1,114.18	10,732.04
16	2.82	1,276.65	6.95	1,079.10	11,102.77
12	2.29	1,278.66	7.97	1,092.04	11,633.02
11	2.51	1,383.45	7.25	1,137.86	11,726.03
13	2.55	1,308.66	8.14	1,104.81	12,325.87
15	2.86	1,426.95	7.39	1,173.30	12,755.23
10	2.53	1,380.57	8.60	1,119.63	13,117.17
19	4.47	1,396.36	8.60	1,174.23	16,335.95

Distance measurements are the average distance differences of the model from the measured values. Energy is the energy penalty remaining after modeling. The FRET restrained columns show the model when it is heavily penalized for failing to satisfy the distance constraints, and the relaxed columns show the same data when the restrained models are unconstrained. Model fit is the sum of the distance × energy products for restrained and relaxed. The starting model for 10–19 was starting model 1 from Table 3.

See also Table S3.

**Figure 5:**
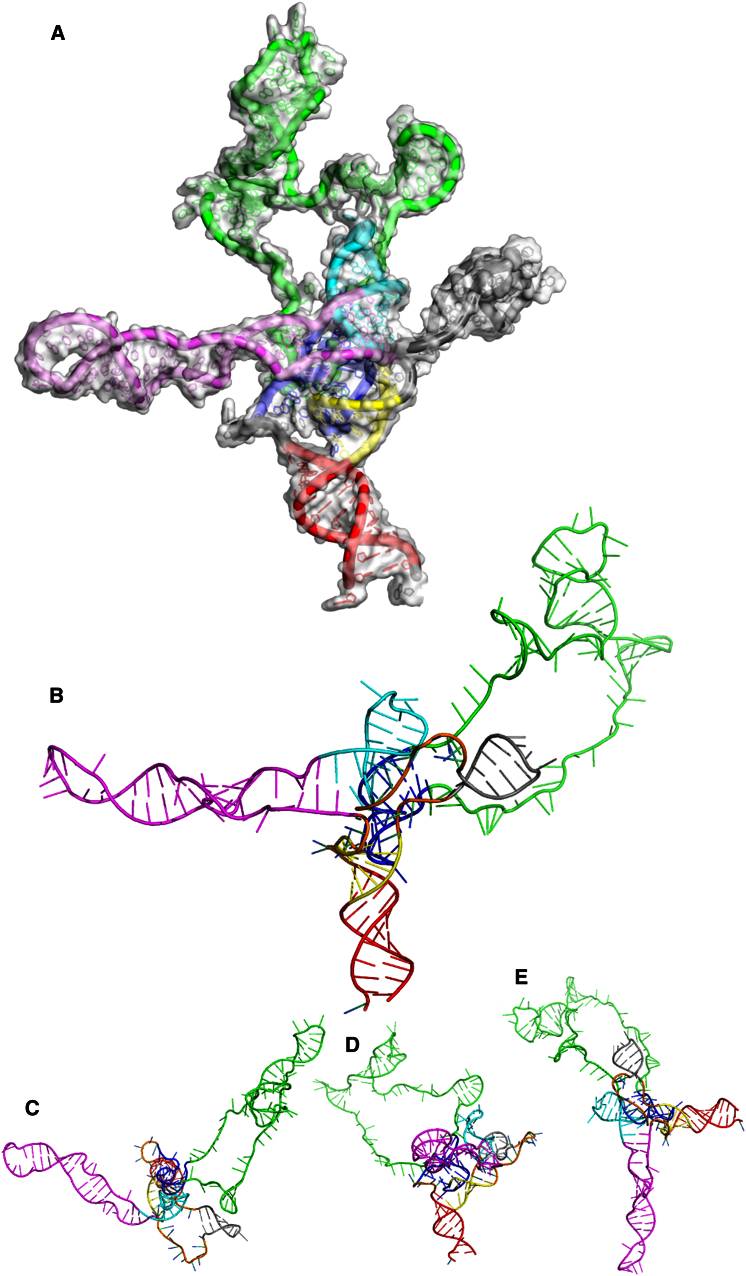
3D Model of nt 104–344 of the HIV-1 5′ UTR RNA Viewed in PyMOL and Colored by Region, Correlating to Figure 3 (A and B) The 3′ extension facilitating PNA annealing and SHAPE probing of the U5-AUG has been removed for clarity. (C–E) Rotated by 90° in the (C) x axis, (D) y axis, and (E) z axis. See also Figure S5 for model variability and sequence conservation.

The structures (10–19) were ranked in the same way as structures 0–9; all but one had a better model fit than structures 0 and 2–9. However, none were better than structure 1 (also derived from starting model r1), so structure 1 was taken as the working model as displayed in Figure 5, with the same color scheme as in Figure 3. The coordinates for the structure have been deposited in the Protein Data Bank under accession number 4AJQ.

To get an estimate for the average structural variation caused by modeling, the 10 refined structures 10–19 (Figure 4B) were aligned with our working model (Figure 5) to minimize the RMSD. The uncertainty of the coordinates (the mean RMSD) was found to be 9.73 Å after removal of the variable PBS and 3′ 345–413 region (Figures S5A and S5B). Hence, this technique can be claimed to achieve a resolution of less than 10 Å. As the flexible RNA in solution has no single structure, a range of structures will variably satisfy physicochemical and measured constraints. We have chosen as our example the most energetically minimized structure.

**Figure 6:**
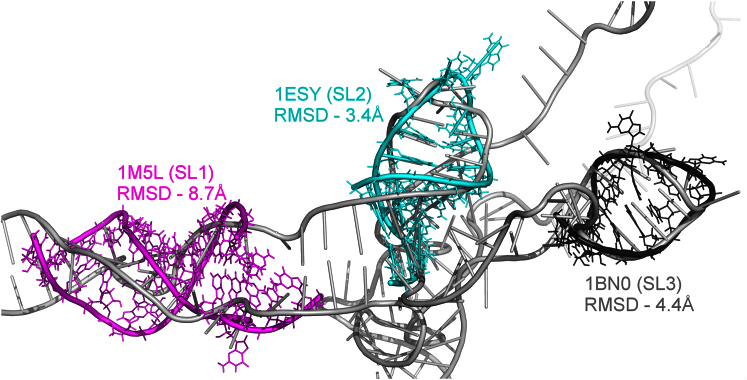
SL1–SL3 Area of Our Working 3D Model of the HIV-1 5′ UTR RNA Showing NMR Structures Aligned using the PyMOL “Align” Function The average distance in angstroms between atoms in the NMR structures and their corresponding atoms in our model is labeled. See also Figure S6.

We assessed the overall quality of our working model in order to consider the confidence assignable to features in the coordinates. Previously solved small subdomain NMR structures for stem loop 1 (SL1) (Greatorex et al., 2002), stem loop 2 (SL2) (Amarasinghe et al., 2000), and stem loop 3 (SL3) (Pappalardo et al., 1998) were superimposed on our structure (Figure 6). After individual optimum alignment, the RMSD between all atoms in the NMR structures and our model was calculated. SL2 and SL3 fit very closely with the predicted model, with RMSD values of 3.4 Å and 4.4 Å, respectively. SL1 fits less well (8.7 Å), although this could be because the NMR structure does not contain the apical loop and may therefore represent a poor comparison. Features in the low-resolution structure here, therefore, represent physically reasonable configurations of the backbone in the target HIV-1 UTR, as the structure adapts to the distance restraints from the SHAPE and FRET experimental analyses. However, given the sparse nature of these restraints, other configurations could be accommodated.

In order to consider the evolutionary likeliness of our working model, we aligned 1,493 HIV-1 sequences and scored nucleotide positions by conservation using the open source software Score Sequence Converter. The colored visualization (Figure S5C) highlights several key areas of high conservation in regions predicted to be structured and low conservation of less structured regions. SL2/SL3 and the primer binding site (PBS), which represent possible ligand binding location, are well conserved. The single-stranded region adjacent to the highly conserved transfer RNA (tRNA) binding PBS is not well conserved, suggesting that the existence of nucleotides to maintain flexibility is more important than the sequence itself.

It may appear unreasonable to expect useful detail in a model of 240 nt based on sparse FRET experimental distance restraints. However, the connectivity of the structure and especially the independently confirmed secondary structure represent strong constraints on the final assembled conformation. Our final model is one of several possible ones that are in the overall space constrained by the FRET distances; the structure represented is that of the lowest energy. Detailed features involving unpaired residues will have less certainty but derive from the energy minimization steps. As a result, these are physically reasonable conformations consistent with the overall fold of the RNA.

## Discussion

In this article, the HIV-1 5′ UTR RNA structure has been predicted from a homogenized monomeric population. Although the accuracy of SmFRET distance measurements can be affected by several different variables such as dye orientation, mobility and shot noise, it has been shown previously (Kalinin et al., 2012) that higher resolution structures can be achieved from low-precision FRET values. The details are driven by the force field of the molecular dynamic prediction but only after the area of structure space is selected by FRET restraints. With few exceptions, our structure is consistent with published biochemically and chemically derived data. The major disparity from that of Watts et al. (2009) is in the PBS region (nt 132–216), which has three helices in our model instead of one, one of which is in the tRNA binding region. This region of the UTR has the least consensus in published structures. Although our SHAPE data in the absence of tRNA^lys^ do not show base pairing within the PBS, the sequence CCCUUUU (150–156) could base pair alternatively with 202–206 GAAAG or 209–214 AAAGG. This metastability, (also seen elsewhere in *psi*; Pappalardo et al., 1998; Greatorex et al., 2002) could explain the disparate results and might facilitate tRNA binding.

The 3D RNA structure has a partly cruciform shape with the two lateral “wings” sweeping backward formed by SL1 and the PBS helix loop. SL3 protrudes forward and upward relative to these, making it accessible to Gag, and the splice donor structure SL2 is tucked behind this. This whole region is thought to adopt alternative structures (Lu et al., 2011a), and the one portrayed with the U5/AUG helix represents a favored model for facilitating genome encapsidation. Thus, the prominence of structures needed for this process and the relatively hidden splice donor make functional sense. Other than the TAR stem loop (Baudin et al., 1993) and the poly-A region (Berkhout, 1996) all the major 5′ UTR structures are present. It is anchored by the well-documented U5/AUG helix. This separates into two widely accepted helices running side by side in antiparallel orientation, one subtending SL1, SL2, and SL3 (Harrison and Lever, 1992) and the other subtending the conserved PBS stem helix (PBS2) (Lu et al., 2011a). To achieve spatial separation of the PBS and SL1, SL2, and SL3, nt 122–125 (UGAC) and 223–226 (GGAG) each form sharp turns in the RNA backbone. These most closely resemble RNA kink turns (Klein et al., 2001) (Figures S6B–S6E). They are also oriented opposite each other in the same plane (Figures S6F and S6G), which suggests a possible protein binding site. It is intriguing that serial mutagenesis of this region disrupting and reforming PBS stem has previously produced inconsistent results; however, re-examining these mutants shows that those disrupting the AG/GA kink-turn motif impair packaging, whereas those maintaining it do not (Clever et al., 2002). The PBS structure itself is open with three helix loop motifs consistent with the Berkhout (1996) model.

Other features, not observable in 2D models, are seen. The nt 220–223 (AGAG) and 230–233 (UCUC) are complementary in sequence and proximal in space (Figure S6H). This may suggest that pairing of 230–233 (UCUC) may be with 220–223 instead of 330–333 (GAGA), or it could represent a switchable conformation. SL2 and SL3 and the PBS region beyond PBS2 form a pocket oriented away from SL1 and the kink turns, which is an attractive candidate for specific Gag binding (Figure S6I).

This investigation of a large virus RNA by FRET has presented much valuable data at this accessible and informative resolution. Information on intramolecular interactions and regions of flexibility provide insight into possible natural ligand binding locations. The technique could be expanded to investigate the change in structure when the sequence is modified, oligonucleotides are added to outcompete tertiary interactions or ligands, natural or designed are added, with potentially therapeutic implications. Fluorophore labeling of ligands will allow further 3D mapping of their binding to the RNA. Structural changes involved in dimerization can also be investigated. It is important to note that, although the species we have solved is purely monomeric, we cannot distinguish whether this is the natural monomer structure or that of a “hemidimer.” Further and more complex analysis of larger RNAs would be needed to define that. However, the solution of either structure can give us critical insights into the functioning of this large viral RNA. The ability to manipulate the system and its versatility give it valuable advantages in RNA structural mapping.

## Experimental Procedures

### RNA Preparation

RNA was transcribed in 20 μl reaction volumes using T7 RNA polymerase (Ambion Megascript T7), according to manufacturer’s instructions, at a template concentration of 15 pM. DNA template was prepared by PCR amplification of pBamH1ΔBglII (Richardson et al., 1995) using primers 5′TAATACGACTCACTATAGGTGTGCCCGTCTGTTG3′ and 5′CTTTCCCCCCTGGCCTTAACC3′. Template was digested for 30 min at 37°C with 3 U TurboDNase (Ambion), and complete removal was verified by electrophoresis of an equivalent concentration of plasmid with and without deoxyribonuclease treatment. RNA was purified on columns (Megaclear, Ambion), according to the manufacturer’s instructions, and eluted with FRET buffer (120 mM KCl, 150 nM CaCl2, 10 mM K_2_HPO_4_/KH_2_PO_4_, 25 mM HEPES, 2 mM EGTA, 5 mM MgCl_2_, pH 7.6) for SHAPE and SmFRET experiments and 1× structure buffer (Ambion) for RNase probing. The integrity and purity of RNAs was determined by native agarose and denaturing PAGE. For SmFRET and RNase mapping experiments, PNA pairs at 10-fold molar excess were annealed to the RNA by overnight incubation at 4°C. Unbound PNAs were removed by gel filtration (Microspin S-400 HR, GE). PNAs were N-terminally labeled (Cambridge Research Biochemicals) with Atto488 (PNA A1- AGAGTCACACAAC, PNA A5- TACTCACCAGT, PNA A4- TCGCTTTCAGGTC) or Atto647 (PNA D6- GCTTAATACTG, PNA D2 TAGAGATTTTCCA, PNA D3- TCGCTTTCAGGTC) via a 1.3-nm-long glycol linker, H_2_N(CH_2_CH_2_O)_2_CH_2_CO. For monomerization, a 10-fold molar excess of LNA (5′GCGCUUC3′; Exiqon) was added, and data were collected for a further 2 hr.

### SHAPE and 2D Modeling

SHAPE was performed and analyzed as described by Kenyon et al. (2011) using primers 5′CTTTCCCCCCTGGCCTTAACC3′ and 5′CAAGCCGAGTCCTGCCTC3′ labeled with 6-FAM, VIC, NED and PET dyes (Applied Biosystems). The following differences in protocol were observed: 5 pmol RNA was used for each reaction, and RNA was probed in FRET buffer, with 4 mM NMIA. Five samples were probed. SHAPE reactivity at each position was scored as +1 if it was above 0.7 and −1 if it was below 0.3; intermediate reactivity was scored as 0.

Structures were modeled using the software RNAstructure (Reuter and Mathews, 2010); SHAPE pseudo-free-energy constraints were used, with a nonconstraining value of −999 at each nucleotide position, unless the cumulative score for the five replicates was positive, in which case their average SHAPE reactivity was used. The region between 191 and 254 was not covered by SHAPE due to dissociation of the reverse transcriptase at the annealed LNA, so biochemical data from previous published work (Harrison and Lever, 1992; Damgaard et al., 1998; Paillart et al., 2004; Watts et al., 2009) were used in this region. Again, only single-stranded restraints were used and only if the majority of studies concurred. 2D structures are displayed using VARNA software (Darty et al., 2009).

### RNase Probing

RNA (2 μg) was digested for 15 min at 37°C with RNase A (50 pg/ml–5 ng/ml), RNase I (5 U/μl–10 U/μl), RNase T1 (1 mU/μl–100 mU/μl), or RNase CV1 (2.5–10 mU/μl), all from Ambion, and precipitated according to the manufacturer’s protocol. Reverse transcription was performed as in Harrison and Lever (1992), using ^33^P-dTTP (Perkin Elmer). Samples were electrophoresed on denaturing 10% polyacrylamide gels, in an equal volume of denaturing loading buffer (Gel Loading Buffer II, Ambion). Gels were transferred to blotting paper, exposed to X-ray films (Kodak Biomax MR) at −80°C, and developed with the Xograph Compact X4 Automatic X-ray Film Processor.

### 3D Topology Atomic Model and Structure Randomization

The 2D arrangement of structural elements was used to produce an initial 3D topology for the native RNA (step 2; Figure 2) using RNA2D3D (Martinez et al., 2008). Standard geometries alone were used, and the experimental FRET distance restraints were not taken into account. In addition, the PNA oligomers were not included. This 3D representation was then normalized using the “generate easy” file in CNS 1.2 (Brunger, 2007), which also generated the molecular topology file. Hydrogen bonding, sugar pucker, base pair and nucleobase planarity, and dihedral angle restraints were derived from the nucleic acids database (Berman et al., 1992).

Pseudorestraints used to randomize starting models had an average length equal to the experimental set but with each actual distance randomly assigned to pairs. Simulated annealing steps with the secondary structure restraints combined with the randomly assigned distance restraints generated the multiple starting conformations with large displacements of secondary structure elements.

### FRET Measurements

A home-built dual-channel confocal fluorescence microscope was used to detect freely diffusing single molecules (Li et al., 2003; Orte et al., 2008). The donor, Atto 488, was excited at 488 nm (Spectra Physics Cyan CDRH, 100 μW), and the acceptor, Atto 647, at 633 nm (He:Ne laser, 25LHP151, Melles Griot). The confocal volume was measured to be 0.4 fl by fluorescence correlation spectroscopy. Donor and acceptor fluorescence were collected through an oil-immersion objective (Nikon Plan Fluor 60×, numerical aperture 1.4) and detected separately by two photon-counting modules (SPCMAQR14, Perkin-Elmer). The outputs of the two detectors were recorded by two computer-implemented multichannel scalar cards (MCS-plus, EG & G, ORTEC). Sample solutions of 50 pM were used to achieve single-molecule detection. All the samples contained 200 μM sodium ascorbate and 0.01% Tween 20 to reduce photobleaching and adsorption of DNA molecules on to the glass surface, respectively. The temperature of the sample was controlled by a thermostage (PE60, Linkam Scientific Instruments). A threshold of 30 counts per millisecond bin for the sum of the donor and acceptor fluorescence signals was used to differentiate single-molecule bursts from the background. A background of between two and three counts per millisecond, obtained from independent measurements of buffer solutions without labeled samples, was subtracted from each burst.

SmFRET measurements were performed in FRET buffer for 2 hr. For monomerization, a 10-fold molar excess of LNA was added 5′GCGCUUC3′ (Exiqon), and data were collected for a further 2 hr. A minimum of five repeat measurements for each pair from different RNA preparations on different occasions reduced condition-specific experimental bias.

### Anisotropy Measurements

Anisotropy was measured for single and pairs of fluorophores as described in Supplemental Experimental Procedures.

### Calculating Distances

Gaussian curves were fitted to frequency/efficiency FRET histograms (Figure S3) using the multiple Gaussian curve fitter in open source QTI plot software. Peaks were assigned as inter-/intramolecular distances based on the change of relative area upon LNA addition. If the relative peak ratio shifted on LNA addition consistently, then the increased peak was assigned as the intramolecular distance. When area shifts contradicted each other, the majority peak was used; if equal, then the repeat with the highest signal:noise ratio was preferred.

The weighted peak efficiency was calculated from the average intensity of signals above the threshold (five times the mean) divided by twice the mean. The interpair distances were calculated using the equations from Gavory et al. (2006), with the Förster distance corrected according to anisotropy. Efficiency tolerances were calculated using the area:height ratio of the weighted mean peak and by transforming into distance tolerances for modeling in the same way as for the interfluorophore distances.

### Simulated Annealing

Ten different starting models were generated by the application of the randomized FRET pairs (step 3; Figure 2), and each was then subject to simulated annealing searches using the standard CNS NMR structure determination script (“anneal.inp” in the standard installation library). Each annealing search (step 4; Figure 2) was run concurrently multiple times with random starting trajectories. Both the initial high temperature stage (2,000 steps at 4,000K) and the following slow cool phase (200 steps at 300K) of the torsional simulated annealing had a van der Waals scale factor of 1.0, and nuclear Overhauser effect (NOE) averaging mode set to “sum.” At the end of each slow-cool annealing search, a further 100 cycles of room temperature minimization were performed. During this stage, any disrupted helices were also forced back into the regular A-form. A final set of 200 cycles of room temperature minimization was applied with the experimental FRET distance restraints specifically removed (step 5; Figure 2).

Table 2 gives restraints applied during the refinements beyond the standard CNS geometric and atomic interaction force-field parameters. The pseudobond distance restraints, including those to prevent the “laddering” distortion of the double-helical sections, are indicated. The relative weighting of these various restraints and of close contacts was determined by the tolerance on the allowed distances and the penalty terms for exceeding these tolerances. The energy penalties appropriate for the CNS refinements were empirically determined but, in practice, departed little from the program defaults as previously applied successfully to RNA structure refinement from NMR restraint data (Nozinovic et al., 2010). Although each FRET experiment used a single pair of fluorescent-labeled PNA probes, all the PNA probe distances were included during refinement as the aim was to produce a single model consistent with all the experimental data.

### 3D Model Selection

The satisfaction of FRET distances was calculated using the PyMOL through-space distance measure. The models were minimized using CNS “minimize.inp,” and the remaining energy penalty was extracted from the output, which allowed our working model to be chosen. A further simulated annealing cycle was then run from the best starting model from the previous step. The similarity of these models was evaluated by aligning models pairwise using the PyMOL “align” function and then storing the results as a similarity matrix. A dendrogram was constructed by neighbor joining from the distance matrix (1 − each value in the similarity matrix) using MEGA (Tamura et al., 2011).

Model resolution was investigated by considering the average distance between atoms in our working model (Figure 5) and those in other simulated annealing structures from the same starting model (Figure S4D) after alignment in PyMOL. The region 132–216 (PBS) was removed for the alignments. This single-stranded region is most likely very flexible; comparing the positions at a particular time is therefore meaningless and will artificially increase the overall mean variation. The artificially added 3′ region was also removed for alignment purposes, as its position is considered unimportant for the understanding of the wild-type structural elements.

The conservation colored model in Figure S5C was from 1,493 aligned HIV-1 sequences scored by Score Sequence Converter software.

The RMSD between NMR structures and our model was calculated using the PyMOL built-in “align” function. Although NMR data were used to assess the model similarity to high resolution data, they were not included in the final model because the necessary minimization step to form a natural and continuous backbone altered both the high resolution structures and decreased the model fit.

## Accession Numbers

The coordinates for the structure have been deposited in the Protein Data Bank under accession number 4AJQ.

## Supplemental Information

Document S1. Figures S1–S6, Tables S1–S3, and Supplemental Experimental Procedures

3D Molecular Model S1. Regions Colored plus Aligned NMR, Related to Figure 5
