# Supplementary material for: Three-Dimensional RNA Structure of the Major HIV-1 Packaging Signal Region
Source: Structure. 2013 Jun 4;21(6):951–62. doi: 10.1016/j.str.2013.04.008 (PMC3690526; doi:10.1016/j.str.2013.04.008)
Supplement: Document S1. Figures S1–S6, Tables S1–S3, and Supplemental Experimental Procedures [file mmc1.pdf]

Structure, Volume *21*

Supplemental Information

Three-Dimensional RNA Structure

# of the Major HIV-1 Packaging Signal Region

James D. Stephenson, Haitao Li, Julia C. Kenyon, Martyn Symmons, Dave Klenerman, and Andrew M.L. Lever

<u>Inventory of Supplemental Information</u>

Figure S1 - Ribonuclease digestion and reverse transcriptase pausing assays to assess RNA 2D structure and PNA effect relates to Figure 3.

Figure S2 - Binding location and anisotropy measurements of annealed fluorophores relates to Table 1.

Figure S3 - FRET histograms showing the frequency of each energy transfer relates to figure 1.

Figure S4 - Searching global and local structure space during simulated annealing relates to figure 4.

Figure S5 Average model variability following simulated annealing and sequence conservation related to figure 5.

Figure S6 A Kink turn and other features of the modelled RNA relates to figure 6.

Table S1 SHAPE reactivity values from probing of the HIV-1 5' untranslated region RNA. relates to figure 3

Table S2 Calculation of weighted efficiencies from signal:noise ratio relates to Table 1

Table S3 Table of the difference between the modelled and measured distances for each fluorophore pair from starting models 0-9 relates to Tables 3 and 4

Supplemental Experimental Procedures

Supplemental Data

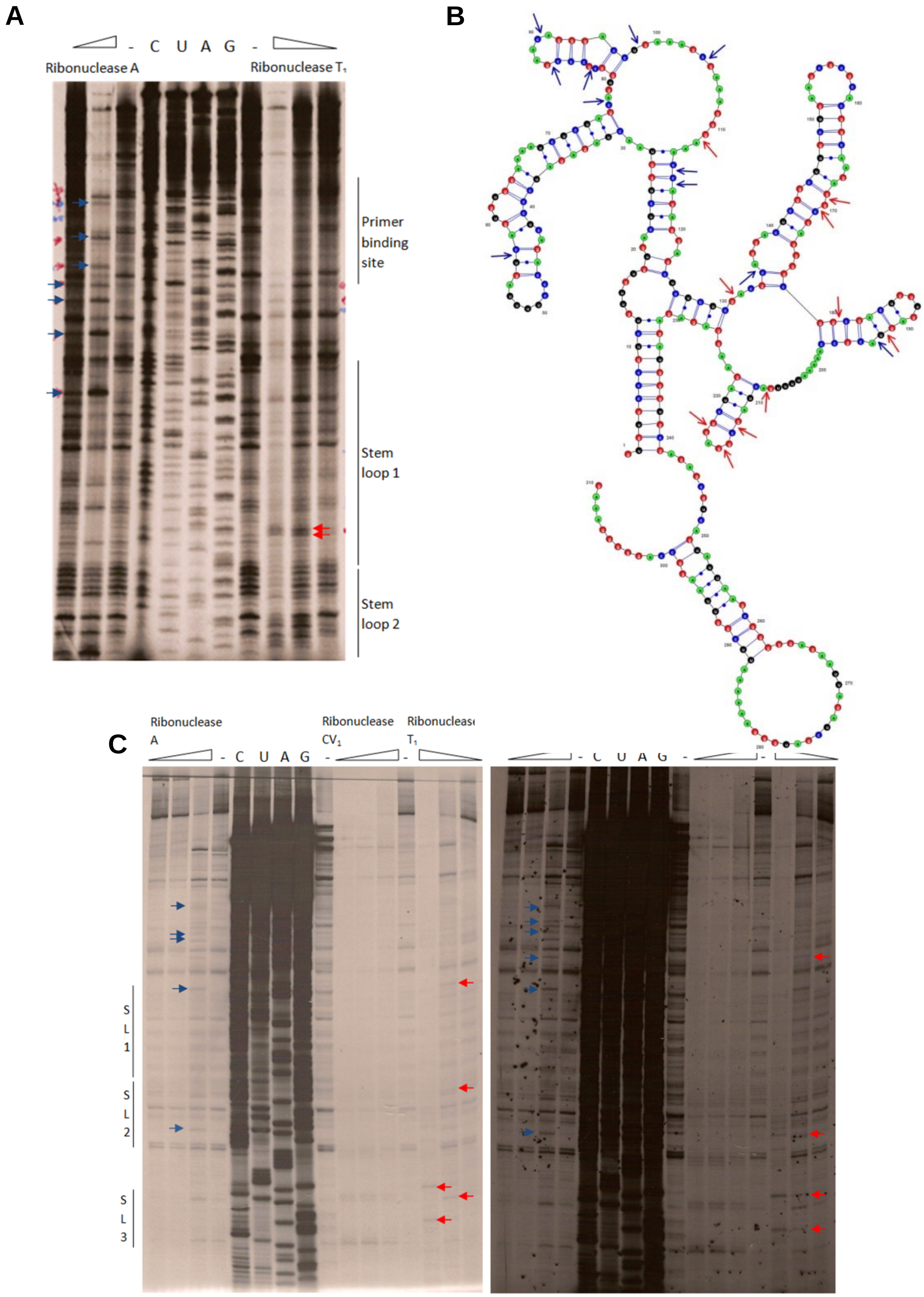

D

PNA	Energy kcal/mol	Hybridization energy kcal/mol	Unfolding PNA kcal/mol	Unfolding RNA kcal/mol	UTR position
A1	-12.6	-20.4	0.1	7.7	116-128
D2	-13.5	-16.9	0.1	3.3	166-177
D3/A4	-17.3	-22.0	0.3	4.4	197-209
A5	-12.3	-16.9	0.1	4.5	286-296
D6	-8.4	-10.2	0	1.8	352-361

E

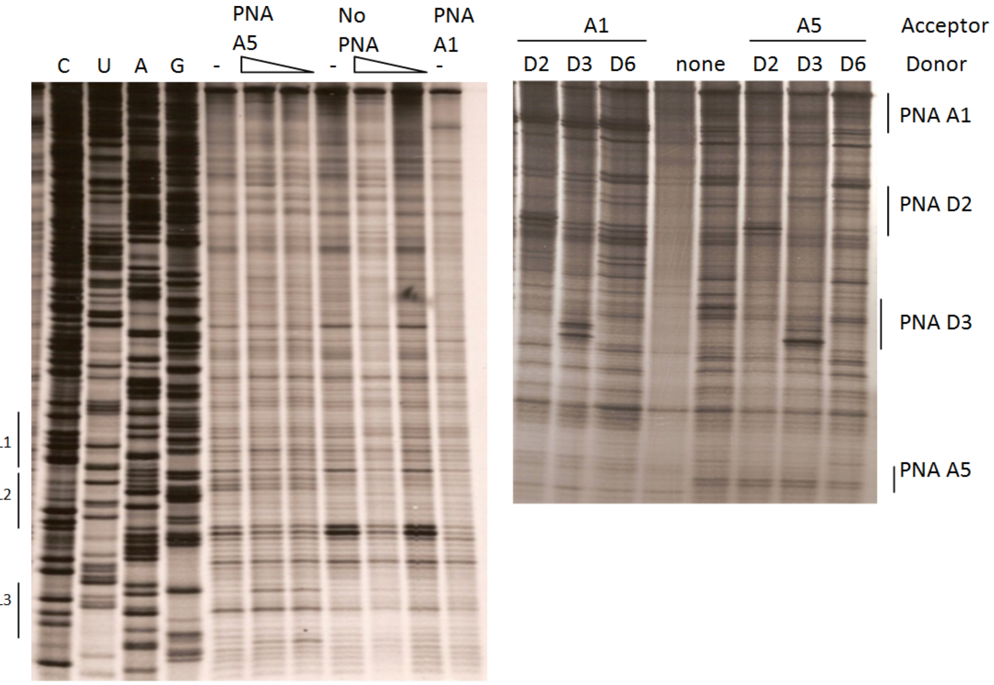

Figure S1. Ribonuclease digestion and reverse transcriptase pausing assays to assess RNA 2D structure and PNA effect.
(A),(C) Biochemical probing (for method see (Harrison et al., 1998a)) using RNase A and T1 showing unpaired regions mapped onto the consensus 2D structure (B)(see also Figure 3). Representative gels shown. Actual data analysed at different exposures to align sequencing ladder accurately with probed lanes. Pause sites of sequence 104-413 were not influenced by the 345-413 segment. Background pausing retains uniformity and is consistent with pausing in other gels from this work which confirm lack of alteration of other pause sites which have been shown to represent structural and sequence motifs within the RNA (for details see (Harrison et al., 1998a).
(D) The Energy column shows the overall energy change after the unfolding of the PNA and RNA is added to the hybridization energy of the PNA to the RNA (Smith et al., 2010). The scoring is based on hybridization free energy and accessibility of the interaction sites in both molecules. Ensemble free energy calculation is realized via the Vienna RNA library (Hofacker et al., 1994).The position of each PNA on the RNA is also shown and can be correlated to the PNA positions on figure 3. (E) PNA binding detected as an additional pause site at the 3' end of the binding site on the RNA. Background pausing retains uniformity when PNA is added which confirms lack of alteration at structural and sequence motifs within the RNA (for details see (Harrison et al., 1998a)).The RNA 2D structure can be seen in figure 3.

A

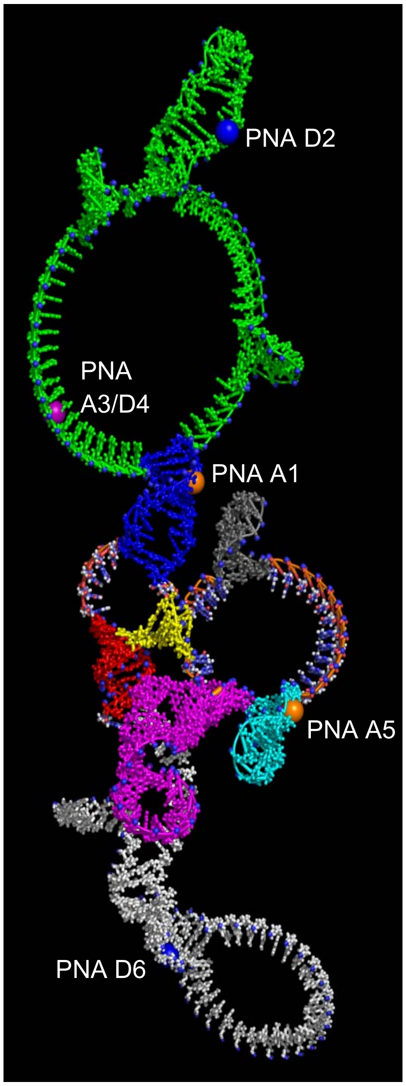

B

Sample	Atto488 anisotropy	Atto647 anisotropy
PNA A1–atto488	0.235 ± 0.005	
PNA A5–atto488	0.309 ± 0.093	
PNA A4–atto488	0.237 ± 0.006	
PNA D6–atto647		0.325 ± 0.082
PNA D2–atto647		0.347 ± 0.032
PNA D3–atto647		0.384 ± 0.043
PNA A5+D2–atto488/647	0.225 ± 0.10	0.215 ± 0.033

Figure S2. Binding location and anisotropy measurements of annealed fluorophores.
(A) 3D representation of the 2D consensus model for the HIV-1 5' untranslated region produced using RNA2D3D (Martinez et al., 2008) and displayed in Pymol. The regions are coloured as in Figure 3 and the fluorophore locations are displayed as spheres on the C1' atoms of the most proximal nucleotide to the fluorophore. The actual experimental PNAs locations are shown in figure 3.
(B) Rotational freedom of the fluorophores was measured by anisotropy experiments, which uncovered the fact that neither the donor or acceptor fluorophores rotate freely probably due to both the linker and having some attraction to the RNA (this is the simplest explanation but not only possible explanation for the increased anisotropy). The orientation factor for the most extreme case (the donor) was corrected. We calculated the orientation of both fluorophore dipoles and hence the dipole orientation factor so as to determine the correct Förster distance to convert the measured FRET efficiencies in donor-acceptor separation. If we assume that the angle of acceptor dipole is 0° because this dye is stuck on the RNA strand then the angle of the donor is 24°.
The Δ factor was calculated to be 1.31 (Supplemental experimental procedure). The Förster distance was therefore corrected by multiplying by the Δ factor which resulted in a change to 6.68nm from the conventional 5.1nm. Distances including anisotropy correction can be seen in Table 1.

PNA A1-D6

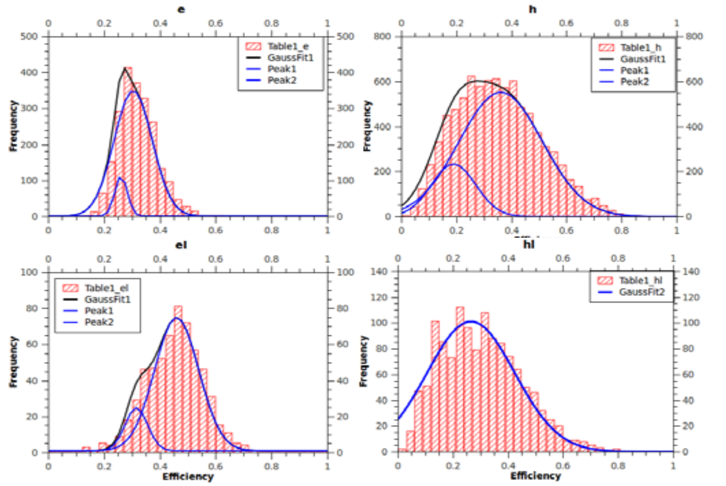

PNA A1-D2

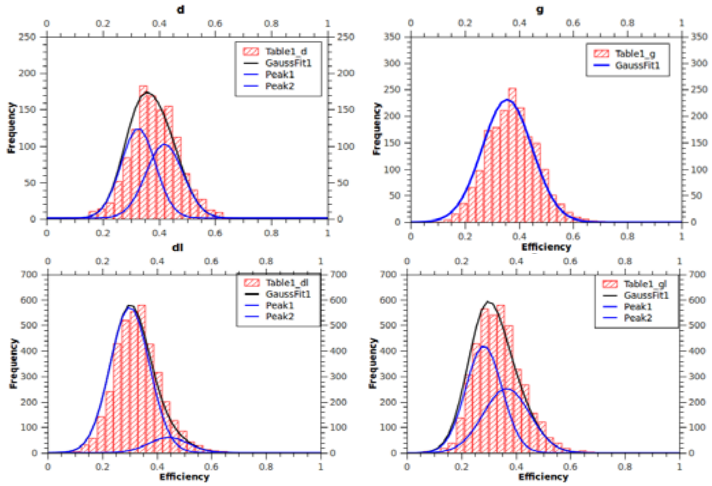

PNA A1-D3

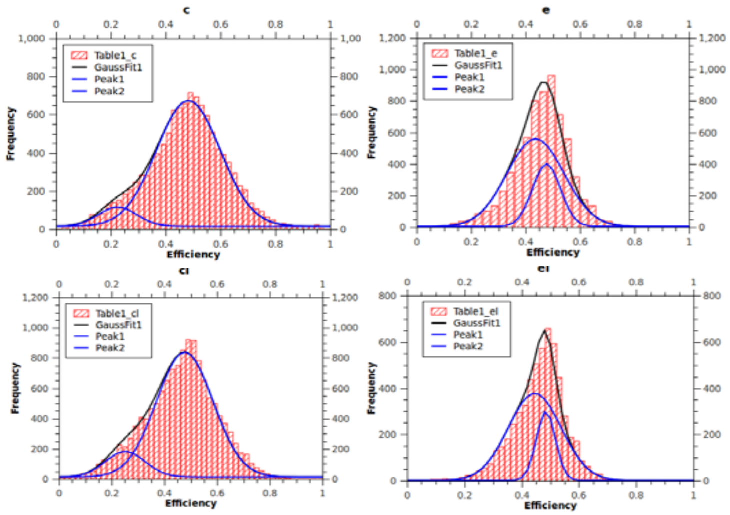

PNA A5-D6

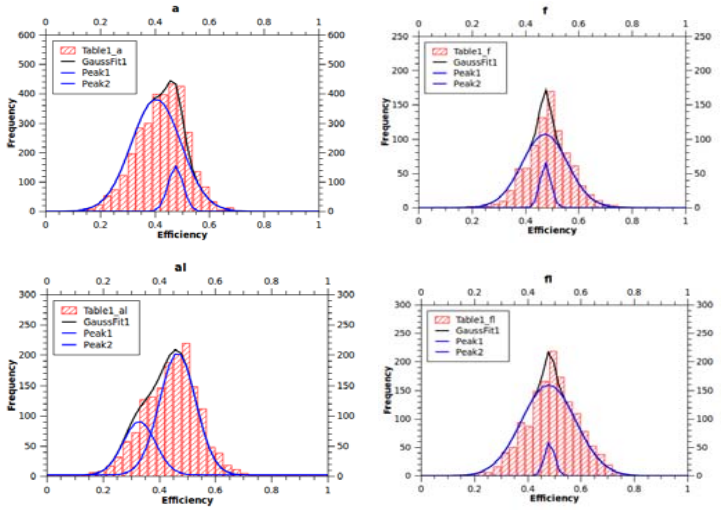

PNA A5-D2

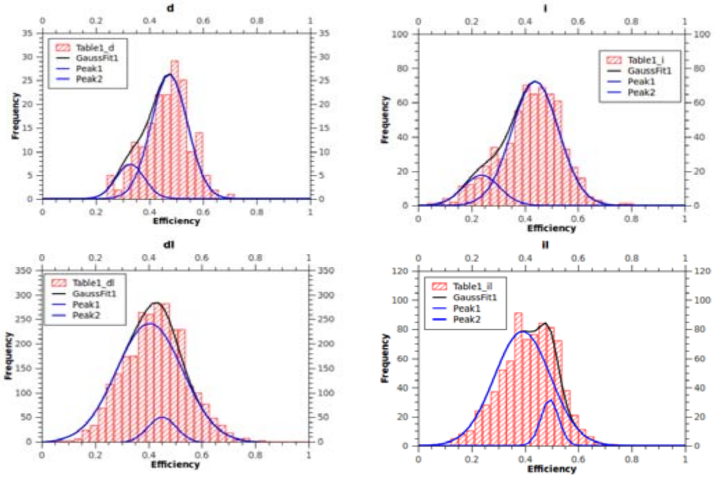

PNA A5-D3

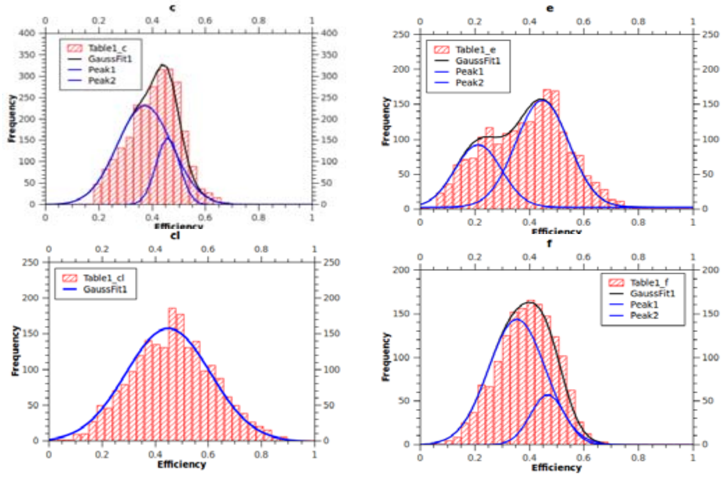

PNA A4-D6

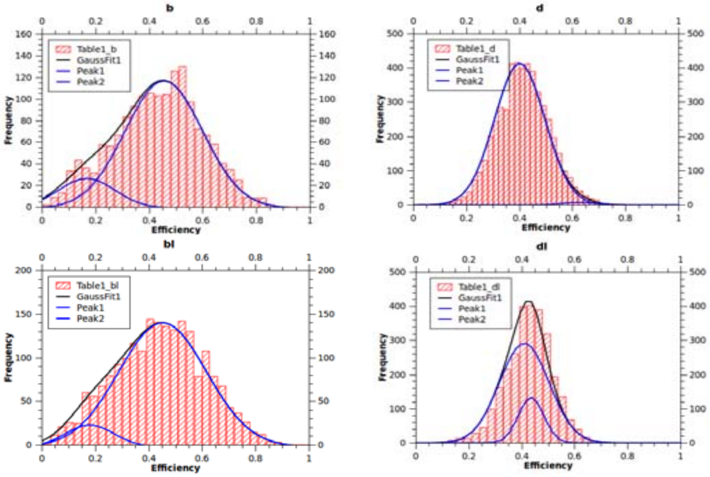

PNA A4-D2

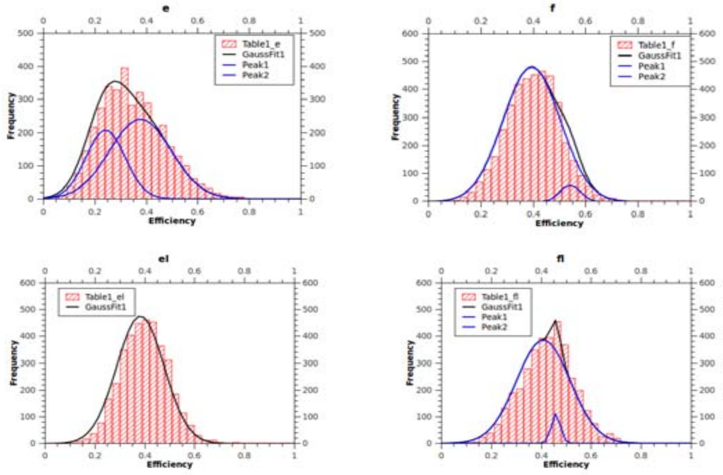

Figure S3. FRET histograms showing the frequency of each energy transfer.
The blue lines show the Gaussian curve predictions from open souce QTI plot software and the black curve is an addition of the Gaussians. Cumulative to RNA flexibility some of the peak width observed was caused by photon shot noise and the flexible linker between PNA and fluorophore resulting in the distances measured essentially being the intersection of spheres of size 1.3nm. The linkers negated the need to incorporate fluorophores into the RNA directly which allowed natural dynamicism of the RNA during FRET measurements. The above data are examples from each pair entitled by their one or two letter experimental references. The numerical data for these examples are found in Table S2. See also figure 1. The full data set is available upon request.

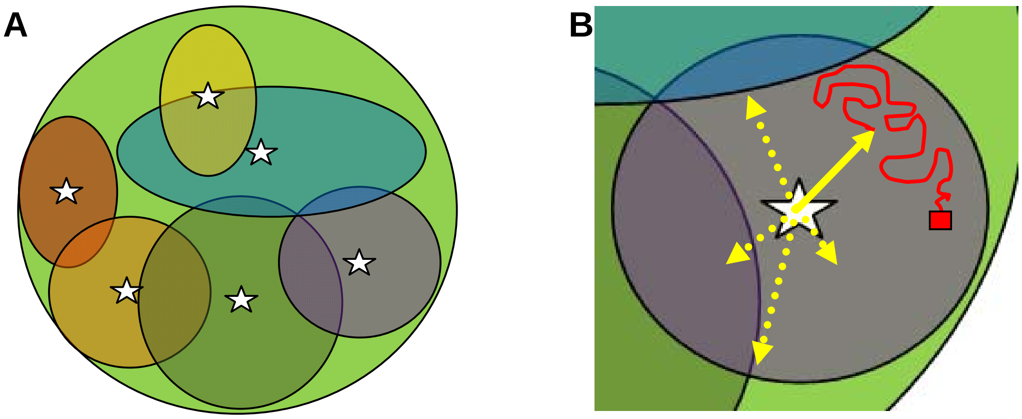

C

Model	0	1	2	3	4	5	6	7	8	9	Total
0	0	36.95	34.41	37.68	30.41	30.27	24.10	28.21	34.00	26.30	282.33
7	28.21	33.18	34.85	38.21	35.58	24.85	33.76	0	36.91	37.90	303.44
6	24.10	43.04	39.73	33.44	26.07	37.31	0	33.76	37.15	29.79	304.38
9	26.30	41.66	37.77	48.80	34.61	34.89	29.79	37.90	32.06	0	323.79
4	30.41	45.85	39.71	29.06	0	45.94	26.07	35.58	39.31	34.61	326.53
2	34.41	23.75	0	34.77	39.71	41.76	39.73	34.85	43.84	37.77	330.59
5	30.27	35.55	41.76	47.70	45.94	0	37.31	24.85	36.97	34.89	335.24
1	36.95	0	23.75	35.81	45.85	35.55	43.04	33.18	41.90	41.66	337.69
8	34.00	41.90	43.84	49.35	39.31	36.97	37.15	36.91	0	32.06	351.49
3	37.68	35.81	34.77	0	29.06	47.70	33.44	38.21	49.35	48.80	354.82

D

Model	10	11	12	13	14	15	16	17	18	19	Total
14	11.03	20.16	11.93	11.63	0	10.99	9.71	12.57	8.32	19.74	116.08
18	12.00	19.93	13.05	13.47	8.32	11.74	9.91	11.95	0	18.20	118.57
16	13.09	19.41	11.27	12.31	9.71	13.69	0	13.33	9.91	20.61	123.32
10	0	20.60	13.78	12.59	11.03	9.86	13.09	14.29	12.00	16.80	124.05
13	12.59	18.19	9.68	0	11.63	13.45	12.31	13.45	13.47	24.84	129.60
15	9.86	23.20	14.76	13.45	10.99	0	13.69	16.38	11.74	17.27	131.34
12	13.78	20.01	0	9.68	11.93	14.76	11.27	15.67	13.05	24.39	134.53
17	14.29	14.13	15.67	13.45	12.57	16.38	13.33	0	11.95	23.24	135.02
11	20.60	0	20.01	18.19	20.16	23.20	19.41	14.13	19.93	31.38	187.01
19	16.80	31.38	24.39	24.84	19.74	17.27	20.61	23.24	18.20	0	196.48

Figure S4. Searching global and local structure space during simulated annealing.
(A) The large green circle represents the total possible structure space for the RNA given the 2D constraints. The stars represent the starting models and their surrounding ovoids represent the searchable structure space available from the starting point due to inherent steric /hindrance. Therefore the more starting points the higher the probability that all possible space is searched. If we assume that the optimum structure is close to the best we have found (model 1) then searching the model 1 structure space more thoroughly is likely to yield a model closer to the ideal. (B) The purple circle represents the searchable structure space from random starting model (r1). The solid yellow arrow indicates the random starting trajectory of the simulated annealing of our current model and the red arrow traces the energy minimization search path across the structure space landscape. The red box represents model 1. We searched the r1 space more thoroughly (as shown in Figure 4B) by assigning different random seeds in order to generate different starting trajectories from r1 and investigate more r1 space (dashed yellow arrows).
Vertically ranked similarity matrices calculated as one minus the pairwise RMSD of each structure when aligned, derived from either (C) random starting models or (D) from the single random starting model “r1” displayed in Figure 5. The aligned structures are shown in Figure 4A/B, structure divergence dendrograms are displayed in Figure 4C/D.

A

Model excluding variable regions	RMSD between our working model and others from the same starting model
0	9.681
1	6.835
2	13.608
3	10.271
4	11.754
5	12.486
6	8.787
7	5.125
8	9.984
9	8.794
Average	9.7325

B

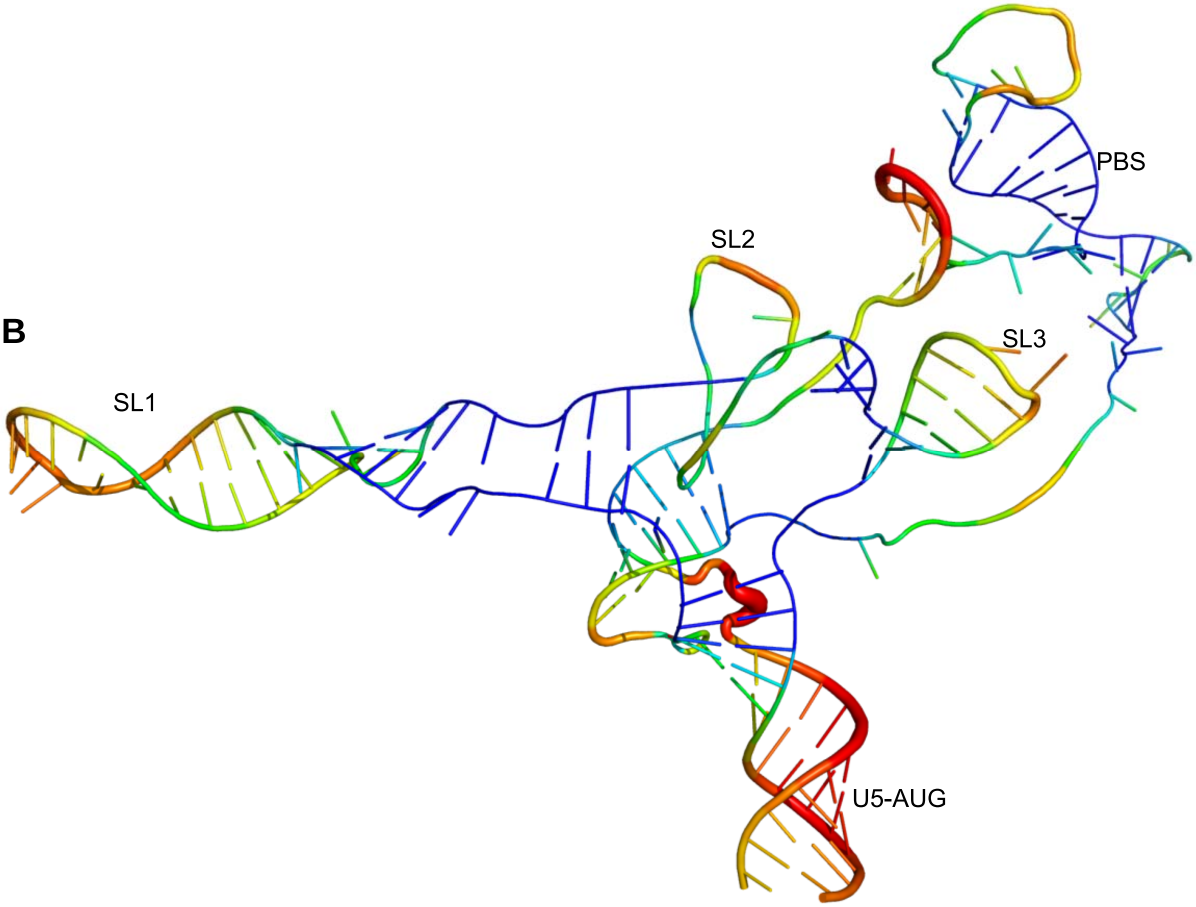

C

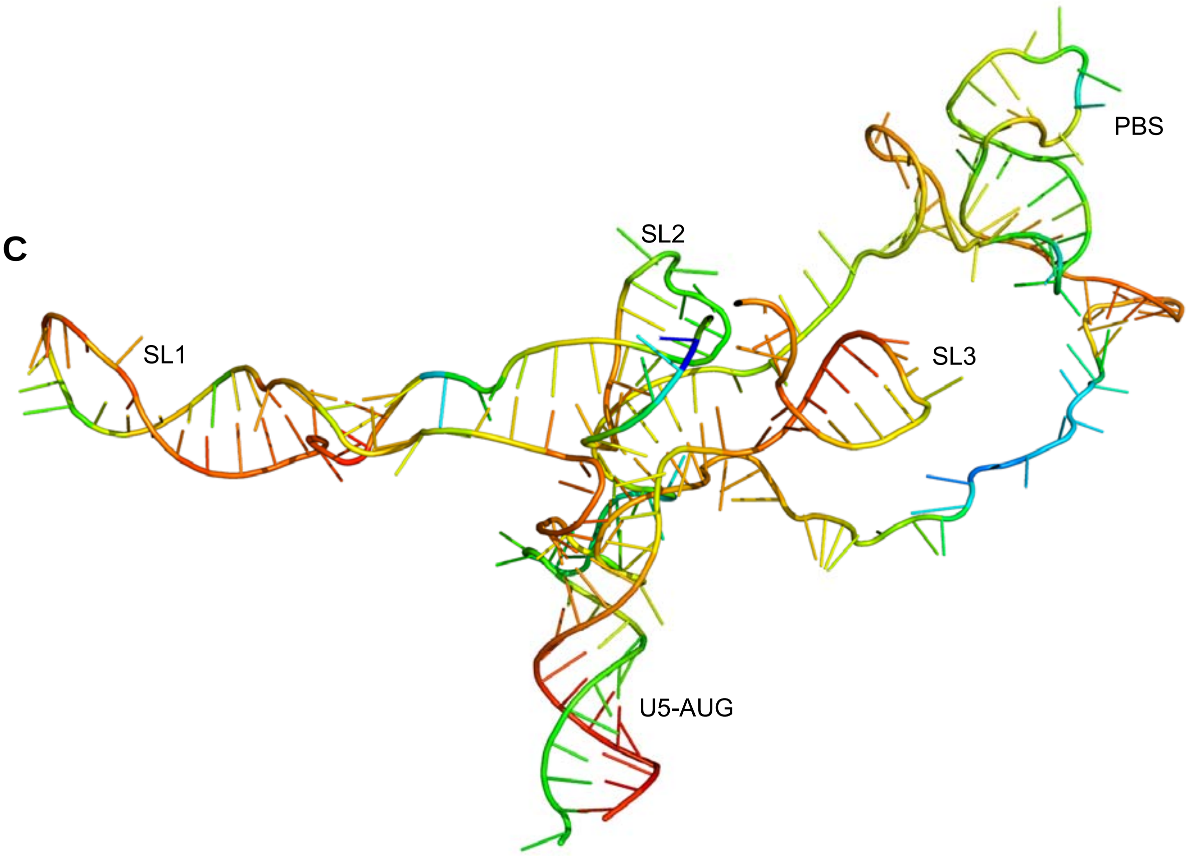

Figure S5 Average model variability following simulated annealing and sequence conservation.
(A) The average distance between atoms in our working model (Figure 5) and those in other simulated annealing structures from the same starting model (Table S4D) after alignment in Pymol. The region 132-216 (PBS) has been removed for the alignments. This single stranded region is most likely very flexible; comparing the positions at a particular time is therefore meaningless and will artificially increase the overall mean variation. The artificially added 3' region has also been removed for alignment purposes as its position is unimportant for the understanding of the wild type structural elements.
(B) 3D structure of the HIV-1 5' untranslated region RNA annotated by structural deviation between models. The molecules backbone is displayed as a continuous width and color scale from thin and blue for low model positional conservation to thick and red for high model positional conservation.
(C) 3D model of the HIV-1 5' untranslated region RNA, nucleotides 104-344, colored by sequence conservation and displayed in Pymol. Coloring is from low conservation (blue) to high conservation (red). See also figure 5.

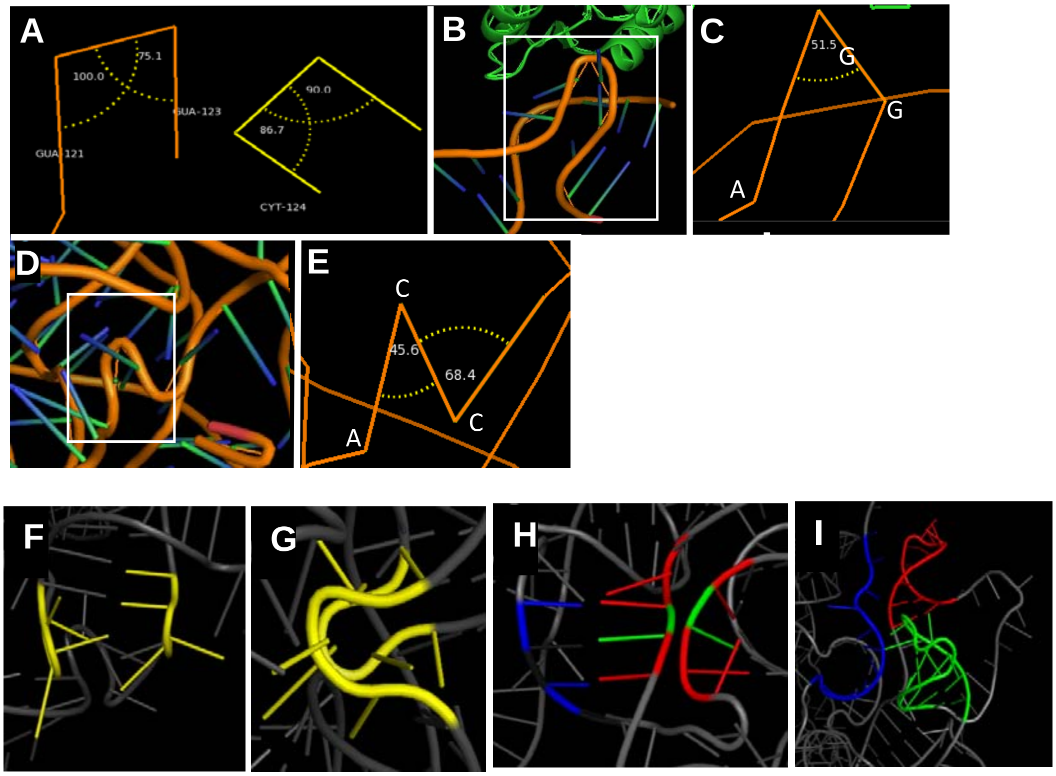

Figure. S6. A Kink turn and other features of the modelled RNA.
Other examples of the sharp, kink turn like, backbone folds in published structures. (A) Pymol ribbon trace between phosphorous atoms including angles between 223-224-225 and 224-225-226 (orange) and 122-123-124 and 123-124-125 (yellow) in our model. (B) White box highlights U4 snRNA (orange backbone (accession 1e7k)) nucleotide positions 133-135. (C) Phosphorus backbone trace and angle between phosphorus atoms of residues 133-131-135. (D) White box highlights nucleotides 272-275 of a self-splicing group-1 intron (accession 1u6b). (E) Phosphorus backbone trace and angle between phosphorus atoms of residues 272-273-274 and of residues 273-274-275.
(F) Highlighted (yellow) tight turns in our working 3D RNA model at positions 123-124 (left) and 224-225 (right). (G) View through turn 224-225 towards turn 123-124. (H) Nucleotides 220-223 (AGAG), far right, and, 230-233 (UCUC) far left, are complementary in sequence and proximal in space in our model. This may suggest that pairing with positions 230-233 (UCUC) may be with 220-223 instead of 330-333 (GAGA) or could represent a switchable conformation. (I) SL2 (green) and SL3 (red) and the PBS region (blue) beyond PBS2 form a pocket orientated away from SL1 and the k-turns which is an attractive candidate for specific Gag binding. See also figure 6.

Position	Sequence	SL1 primer					Repeat score					Total score	SHAPE reactivity						
104	G		0.662										-999						
105	U		0.084										-999						
106	G	0.241	1.267	0.338									-999						
107	U	0.731	0.579	0.649									-999						
108	G	0.299	0.043	0.205	0.93								-999						
109	C	0.088	0.726	0.702	0.288	0.934	-1	1	1	-1	1	1	0.548						
110	C	0.193	0.323	0.077	0.479	0.605	-1	0	-1	0	0	-2	-999						
111	C	0.264	0.708	0.168	0.496	0.12	-1	1	-1	0	-1	-2	-999						
112	G	1.447	0.856	1.055	0.229	0.925	1	1	1	-1	1	3	0.902						
113	U	0.594	0.302	0.387	0.359	0.967	0	0	0	0	1	1	0.522						
114	C	0.167	0.243	0.15	0.355	0.877	-1	-1	-1	0	1	-2	-999						
115	U	0.431	0.141	0.28	0.446	0.657	0	-1	-1	0	0	-2	-999						
116	G	0.583	0.535	0.546	0.719	1.018	0	0	0	1	1	2	0.68						
117	U	0.16	0.033	-0.057	0.361	0.557	-1	-1	-1	0	0	-3	-999						
118	U	0.356	0.578	0.494	0.833	0.677	0	0	0	1	0	1	0.587						
119	G	0.03	-0.018	0.002	0.372	0.59	-1	-1	-1	0	0	-3	-999						
120	U	0.411	0.549	0.459	0.732	0.755	0	0	0	1	1	2	0.581						
121	G	0.176	0.524	0.374	0.713	0.964	-1	0	0	1	1	1	0.55						
122	U	0.146	0.689	0.225	0.619	0.577	-1	0	-1	0	0	-2	-999						
123	G	0.906	0.652	0.8	0.505	0.77	1	0	1	0	1	3	0.727						
124	A	0.072	0.198	0.108	0.247	0.293	-1	-1	-1	-1	-1	-5	-999						
125	C	0.759	0.721	0.927	0.445	0.699	1	1	1	0	0	3	0.71						
126	U	0.081	0.399	0.479	0.665	0.481	-1	0	0	0	0	-1	-999						
127	C	-0.132	0.107	0.092	0.55	0.587	-1	-1	-1	0	0	-3	-999						
128	U	0.669	0.584	-0.039	0.545	0.156	0	0	-1	0	-1	-2	-999						
129	G	-0.262	0.525	0.577	0.72	0.633	-1	0	0	1	0	0	-999						
130	G	0.348	0.521	0.532	1.066	1.042	0	0	0	1	1	2	0.702						
131	U	0.086	0.502	0.206	0.564	0.575	-1	0	-1	0	0	-2	-999						
132	A	1.128	0.402	0.81	0.265	0.677	1	0	1	-1	0	1	0.656						
133	A	0.841	0.123	0.67	0.497	0.556	1	-1	0	0	0	0	-999						
134	C	0.107	0.211	0.416	0.87	0.668	-1	-1	0	1	0	-1	-999						
135	U	0.165	0.006	0.319	0.305	0.171	-1	-1	0	0	-1	-3	-999						
136	A	-0.075	0.093	0.083	0.308	0.447	-1	-1	-1	0	0	-3	-999						
137	G	0.194	0.019	0.157	0.578	0.334	-1	-1	-1	0	0	-3	-999						
138	A	-0.002	0.57	0.257	0.598	0.503	-1	0	-1	0	0	-2	-999						
139	G	0.287	0.796	0.569	0.569	0.656	-1	1	0	0	0	0	-999						
140	A	0.46	1.035	0.796	0.207	0.666	0	1	1	-1	0	1	0.633						
141	U	0.61	0	0.532	0.263	0.445	0	-1	0	-1	0	-2	-999						
142	C	0.411	0.08	0.449	0.315	0.305	0	-1	0	0	0	-1	-999						
143	C	0.242	0.254	0.294	0.631	0.398	-1	-1	-1	0	0	-3	-999						
144	C	1.843	0.271	0.943	0.265	0.405	1	-1	1	-1	0	0	-999						
145	U	0.095	-0.03	0.358	1.011	0.506	-1	-1	0	1	0	-1	-999						
146	C	0.448	0.167	0.428	0.689	0.336	0	-1	0	0	0	-1	-999						
147	A	0.059	0.33	0.098	0.454	0.311	-1	0	-1	0	0	-2	-999						
148	G	0.537	0.67	0.492	0.276	0.405	0	0	0	-1	0	-1	-999						
149	A	0.376	0.86	0.39	0.016	0.099	0	1	0	-1	-1	-1	-999						
150	C	0.782	0.833	0.539	0.421	0.048	1	1	0	0	-1	1	0.525						
151	C	0.681	0.458	0.46	0.575	0.331	0	0	0	0	0	0	-999						
152	C	0.202	0.039	0.2	0.322	0.355	-1	-1	-1	0	0	-3	-999						
153	U	0.745	0.569	0.554	0.845	0.617	1	0	0	1	0	2	0.666						
154	U	0.836	0.453	0.679	0.631	0.534	1	0	0	0	0	1	0.626						
155	U	0.2	0.139	0.398	0.769	0.681	-1	-1	0	1	0	-1	-999						
156	U	0.242	0.174	0.365	0.675	0.594	-1	-1	0	0	0	-2	-999						
157	A	1.042	0.855	0.876	0.991	0.438	1	1	1	1	0	4	0.84						
158	G	2.478	0.193	1.729	-0.144	0.497	1	-1	1	-1	0	0	-999						
159	U	0.146	0.256	0.253	0.479	0.581	-1	-1	-1	0	0	-3	-999						
160	C	0.48	0.312	0.585	0.617	0.58	0	0	0	0	0	0	-999						
161	A	0.498	0.066	0.225	0.308	0.102	0	-1	-1	0	-1	-3	-999						
162	G	-0.004	0.224	0.413	0.583	0.585	-1	-1	0	0	0	-2	-999						
163	U	0.612	0.661	1.197	0.745	0.955	0	0	1	1	1	3	0.834						
164	G	-0.447	0.122	-0.257	1.035	-0.182	-1	-1	-1	1	-1	-3	-999						
165	U	0.621	0.312	0.275	0.244	0.317	0	0	-1	-1	0	-2	-999						
166	G	0.201	0.632	0.818	0.662	0.541	-1	0	1	0	0	0	-999						
167	G	0.108	0.137	0.259	0.458	0.613	-1	-1	-1	0	0	-3	-999						
168	A	0.284	0.261	0.38	0.782	0.598	-1	-1	0	1	0	-1	-999						
169	A	0.323	0.205	0.319	0.862	0.532	0	-1	0	1	0	0	-999						
170	A	0.622	0.627	0.661	0.862	0.491	0	0	0	1	0	1	0.653						
171	A	0.882	0.988	1.284	0.25	0.074	1	1	1	-1	-1	1	0.696						
172	U	0.353	0.57	0.412	0.306	0.4	0	0	0	0	0	0	-999						
173	C	1.308	1.139	1.356	0.189	0.534	1	1	1	-1	0	2	0.905						
174	U	0.894	0.547	0.916	0.628	0.161	1	0	1	0	-1	1	0.629						
175	C	0.056	0.041	0.101	0.652	0.13	-1	-1	-1	0	-1	-4	-999						
176	U	0.149	0.307	0.963	1.097	1.12	-1	0	1	1	1	2	0.727						
177	A	3.208	0.445		0.046	0.004							-999						
178	G	0.568	0.275		0.869	0.595							-999						
179	C	0.024	0.134		0.018	0.8							-999						
180	A	0.511			0.017	0.803							-999						
181	G	-0.122			0.696	0.475							-999						
182	U	0.755			0.587	0.585							-999						
183	G	0.28			-0.208	-0.152							-999						
184	G	-0.145			0.591	0.066							-999						
185	C	0.391			0.578	0.688							-999						
186	G	0.816			0.271	0.152							-999						
187	C	-0.045			0.414	0.614							-999						
188	C	2.13			0.784	0.031							-999						
189					0.786	0.728							-999	States based on previous data					Total
190					-0.264								-999	Har	Da	Pai	Watt		
191					0.497								1	-2	-2	-2	0.25	2	-4
192													-999	-2	-2	-2	0.301	0	-6
193													-999	-2	-2	2	0.145	2	0
194													1	-2	-2	-2	0	2	-4
195													-999	2	-2		0.021	2	2
196													-999		-2		0.094	2	0
197													-999		-2		0	2	0
198													-999	-2		-2	0	2	-2
199													-999			2	0.01	2	4
200													-999				0.557	0	0
201													-999				0.786	-2	-2
202													-999	-2			0.528	0	-2
203													1	-2	-1	-2	0.667	0	-5
204													1	-2	-1	-2	0.79	-2	-7
205													1	-2	-1		0.827	-2	-5
206													1	-2			0.891	-2	-4
207													-999			-2	0.629	0	-2
208													-999	-2			0.474	0	-2
209													1	-2	-1	-2	0.837	-2	-7
210													1	-2	-1	-2	0.959	-2	-7
211													1	-2			1.064	-2	-4
212													1	-2	-2		1.061	-2	-6
213													1	-2	-2		0.344	0	-4
214													1	-2	-2		0.947	-2	-6
215													1	-2	-2	-2	1.002	-2	-8
216													1	-2		-2	1.116	-2	-6
217													-999			-2	0.334	0	-2
218													-999			2	0.074	2	4
219													-999			2	0.016	2	4
220													-999			2	0.055	2	4
221													-999	-2			0.123	2	0
222													-999	2		-1	0.185	2	3
223													-999				0.226	2	2
224													-999				0.584	0	0
225													-999			-2	0.473	0	-2

226													-999				0.61	0	0
227													1			-1	0.575	0	-1
228													-999	2			0.317	0	2
229													-999			-2	0.036	2	0
230													-999				0.002	2	2
231													-999			-1	0.007	2	1
232													-999				0	2	2
233													-999			-1	0.044	2	1
234													-999				0.235	2	2
235													-999			-2	0.419	0	-2
236													-999			2	0.213	2	4
237													-999				0.24	2	2
238													-999	2		-2	0.582	0	0
239													-999			-2	0.637	0	-2
240													-999				1.861	-2	-2
241													-999				1.594	-2	-2
242													1	-2		-2	0.845	-2	-6
243													-999			2	0.19	2	4
244													-999	2			0.052	2	4
245													-999			2	0.043	2	4
246													-999				0.129	2	2
247													-999				0.519	0	0
248													-999	2		2	0.094	2	6
249													-999				0.031	2	2
250	Sequence			8B primer					Repeat score			Total score	-999				0.015	2	2
251				0.343									-999	-2			0	2	0
252				-0.295									-999			2	0	2	4
253				0.058									-999	2			0	2	4
254				0.177									-999	-2			0.216	2	0
255				0.29									1						
256				-0.498									1						
257				-0.084									1						
258				0.151									1						
259				0.056									1						
260	C	0.391		-0.193	0.937								1						
261	G	0.529	0.636	0.106	0.077	0.105	0	0	-1	-1	-1	-3	-999						
262	C	0.256	0.35	0.157	-0.002	-0.138	-1	0	-1	-1	-1	-4	-999						
263	A	0.286	0.125	0.057	1.043	-0.032	-1	-1	-1	1	-1	-3	-999						
264	C	1.167	0.612	-0.453	4.94	0.105	1	0	-1	1	-1	0	-999						
265	G	2.449	0.285	-0.276	5.127	0.362	1	-1	-1	1	0	0	-999						
266	G	14.585	-0.432	2.214	1.172	7.186	1	-1	1	1	1	3	4.945						
267	C	11.304	-0.356	3.331	0.583	-0.019	1	-1	1	0	-1	0	-999						
268	A	0.677	0.167	0.46	0.576	0.733	0	-1	0	0	1	0	-999						
269	A	0.713	-0.064	0.441	0.243	0.624	1	-1	0	-1	0	-1	-999						
270	G	0.721	0.418	0.239	0.556	0.207	1	0	-1	0	-1	-1	-999						
271	A	3.569	0.927	0.792	-0.017	0.166	1	1	1	-1	-1	1	1.087						
272	G	1.693	1.879	0.591	0.137	0.389	1	1	0	-1	0	1	0.938						
273	G	0.239	0.815	0.804	-0.249	0.246	-1	1	1	-1	-1	-1	-999						
274	C	0.303	-0.225	0.454	0.125	0.077	0	-1	0	-1	-1	-3	-999						
275	G	0.401	-0.094	0.007	0.184	0.163	0	-1	-1	-1	-1	-4	-999						
276	A	0.465	0.206	0.542	0.126	0.04	0	-1	0	-1	-1	-3	-999						
277	G	0.622	0.197	0.559	0.122	0.097	0	-1	0	-1	-1	-3	-999						
278	G	0.32	0.422	0.773	0.112	0.236	0	0	1	-1	-1	-1	-999						
279	G	0.384	0.171	0.485	0.098	0.343	0	-1	0	-1	0	-2	-999						
280	G	0.489	-0.069	0.618	-0.434	0.733	0	-1	0	-1	1	-1	-999						
281	C	0.282	0.399	1.01	-1.563	-1.113	-1	0	1	-1	-1	-2	-999						
282	G	0.844	0.327	1.592	-0.436	-0.277	1	0	1	-1	-1	0	-999						
283	G	0.973	0.037	0.72	0.274	-0.016	1	-1	1	-1	-1	-1	-999						
284	C	0.11	0.35	0.616	0.466	0.058	-1	0	0	0	-1	-2	-999						
285	G	0.334	0.181	0.651	0.201	0.267	0	-1	0	-1	-1	-3	-999						
286	A	0.295	0.131	0.562	0.727	0.693	-1	-1	0	1	0	-1	-999						
287	C	0.296	-0.007	0.884	0.347	0.225	-1	-1	1	0	-1	-2	-999						
288	U	-0.056	0.229	0.858	0.072	0.394	-1	-1	1	-1	0	-2	-999						
289	G	0.777	0.138	1.558	0.081	-0.037	1	-1	1	-1	-1	-1	-999						
290	G	11.133	1.107	10.434	0.242	0.38	1	1	1	-1	0	2	4.659						
291	U	0.536	0.346	0.631	0.207	0.327	0	0	0	-1	0	-1	-999						
292	G	0.496	-0.152	1.01	0.045	0.022	0	-1	1	-1	-1	-2	-999						
293	A	0.577	-0.055	0.658	-0.113	0.074	0	-1	0	-1	-1	-3	-999						
294	G	0.692	0.841	0.304	0.628	0.37	0	1	0	0	0	1	0.567						
295	U	0.201	0.447	0.03	0.218	0.486	-1	0	-1	-1	0	-3	-999						
296	A	3.345	0.365	2.484	0.304	0.151	1	0	1	0	-1	1	1.33						
297	C	0.307	0.286	0.378	0.078	0.105	0	-1	0	-1	-1	-3	-999						
298	G	0.614	-0.026	-0.03	0.395	0.945	0	-1	-1	0	1	-1	-999						
299	C	5.234	0.514	5.103	0.155	0.327	1	0	1	-1	0	1	2.267						
300	C	-0.324	0.423	1.883	0.27	0.192	-1	0	1	-1	-1	-2	-999						
301	A	0.232	-0.232	0.519	0.589	0.241	-1	-1	0	0	-1	-3	-999						
302	A	0.261	0.49	0.539	0.834	0.491	-1	0	0	1	0	0	-999						
303	A	-0.116	0.321	0.512	0.971	0.903	-1	0	0	1	1	1	0.518						
304	A	0.45	0.313	0.584	0.7	1.559	0	0	0	1	1	2	0.721						
305	A	-0.131	0.406	0.396	0.345	1.074	-1	0	0	0	1	0	-999						
306	U	0.193	0.468	0.636	0.481	0.624	-1	0	0	0	0	-1	-999						
307	U	-0.091	0.041	0.521	0.148	0.141	-1	-1	0	-1	-1	-4	-999						
308	U	2.7	-0.065	0.703	0.564	0.638	1	-1	1	0	0	1	0.908						
309	U	0.966	0.256	1.084	0.331	0.193	1	-1	1	0	-1	0	-999						
310	G	0.755	0.325	0.607	1.036	1.26	1	0	0	1	1	3	0.797						
311	A	0.737	0.355	1.027	0.57	0.002	1	0	1	0	-1	1	0.538						
312	C	0.857	0.486	0.796	0.797	0.783	1	0	1	1	1	4	0.744						
313	U	0.719	0.641	0.629	0.239	0.512	1	0	0	-1	0	0	-999						
314	A	0.44	0.544	0.341	0.127	0.636	0	0	0	-1	0	-1	-999						
315	G	0.44	0.442	0.306	0.527	0.282	0	0	0	0	-1	-1	-999						
316	C	0.616	0.693	0.421	0.188	0.215	0	0	0	-1	-1	-2	-999						
317	G	0.741	0.276	0.469	0	0.012	1	-1	0	-1	-1	-2	1						
318	G	0.355	0.458	0.279	0.16	0.241	0	0	-1	-1	-1	-3	1						
319	A	0.277	-0.067	0.249	-0.122	0.203	-1	-1	-1	-1	-1	-5	1						
320	G	0.166	0.278	0.067	0.351	0.099	-1	-1	-1	0	-1	-4	1						
321	G	0.511	0.437	0.345	0.575	0.626	0	0	0	0	0	0	-999						
322	C	0.251	0.506	0.469	0.52	0.876	-1	0	0	0	1	0	-999						
323	U	0.068	0.417	-0.062	0.434	0.72	-1	0	-1	0	1	-1	-999						
324	A	-0.06	0.175	-0.003	0.19	0.228	-1	-1	-1	-1	-1	-5	-999						
325	G	0.43	0.621	-0.944	0.298	0.346	0	0	-1	-1	0	-2	-999						
326	A	-0.025	0.399	0.404	0.194	0.083	-1	0	0	-1	-1	-3	-999						
327	A	0.225	-0.013	0.635	-0.067	-0.016	-1	-1	0	-1	-1	-4	-999						
328	G	-0.118	0.117	-0.157	0.021	0.113	-1	-1	-1	-1	-1	-5	-999						
329	G	0.085	0.242	0.289	0.025	0.266	-1	-1	-1	-1	-1	-5	-999						
330	A	0.238	0.216	0.302	0.023	0.202	-1	-1	0	-1	-1	-4	-999						
331	G	1.564	0.193	0.922	0.358	0.272	1	-1	1	0	-1	0	-999						
332	A	0.95	0.727	0.632	0.685	-0.076	1	1	0	0	-1	1	0.583						
333	G	0.562	1.982	0.396	0.692	0.469	0	1	0	0	0	1	0.82						
334	A	0.164	1.038	0.346	0.72	0.699	-1	1	0	1	0	1	0.593						
335	G	0.318	-0.065	0.445	0.045	0.598	0	-1	0	-1	0	-2	-999						
336	A	0.025	0.23	0.013	0.527	0.942	-1	-1	-1	0	1	-2	-999						
337	U	0.44	0.248	-0.012	-0.044	0.163	0	-1	-1	-1	-1	-4	-999						
338	G	0.192	-0.041	0.105	-0.105	0.286	-1	-1	-1	-1	-1	-5	-999						
339	G	0.386	0.093	0.204	0.207	1.361	0	-1	-1	-1	1	-2	-999						
340	G	0.665	-0.069	0.616	0.48	0.087	0	-1	0	0	-1	-2	-999						
341	U	0.649	0.671	0.586	-0.083	0.68	0	0	0	-1	0	-1	-999						
342	G	0.621	1.041	0.558	1.006	0.565	0	1	0	1	0	2	0.758						
343	C	0.321	0.337	0.409	0.303	0.077	0	0	0	0	-1	-1	-999						
344	G	0.459	0.321	0.348	0.448	0.525							-999						
345	A	0.954	-0.136	0.661	0								-999						
346	G	0.523	0.543	0.438	0.412								-999						
347	A	-0.08	0.404	-0.143	0.066								-999						
348	G	-0.088	-0.017	-0.522	0.732								-999						

349	C	0.215	0.312	1.065	0.039								-999						
350	G	0.173	0.038	0.046	0.328								-999						
351	U	0.254	-0.062	-0.18	2.461								-999						
352		0.734	0.811	-0.237									-999						
353		0.439	0.408	0.627									-999						
354		0.074	0.637	0.064									-999						
355		1.271	0.693	1.138									-999						
356		0.184	-0.29	0.138									-999						
357		-0.233	0.488	0.555									-999						
358		0.345	0.906	0.13									-999						
359		0.018	0.256	0.136									-999						
360		0.106	0.943	-0.82									-999						
361		-0.31	0.675	0.663									-999						
362		0.326	0.278	-0.387									-999						
363		-0.57	0.948	0.332									-999						
364		-0.367	0.258	0.242									-999						
365		0.784	1.102	0.351									-999						
366		0.419	0.988	0.234									-999						
367		0.513	-0.03	0.181									-999						
368		1.017	-0.201	-0.132									-999						
369			0.569	0.916									-999						
370			0.028	1.561									-999						
371			2.282	3.877									-999						
372			3.436	6.954									-999						
373				0.094									-999						
374													-999						
375													-999						
376													-999						
377													-999						
378													-999						
379													-999						
380													-999						
381													-999						
382													-999						
383													-999						
384													-999						
385													-999						
386													-999						
387													-999						
388													-999						
389													-999						
390													-999						
391													-999						
392													-999						
393													-999						
394													-999						
395													-999						
396													-999						
397													-999						
398													-999						
399													-999						
400													-999						
401													-999						
402													-999						
403													-999						
404													-999						
405													-999						
406													-999						
407													-999						
408													-999						
409													-999						
410													-999						
411													-999						
412													-999						
413													-999						

Table S1. SHAPE reactivity values from probing of the HIV-1 5' untranslated region RNA. SHAPE reactivities were quantitated as in (Watts et al., 2009) and coloured by value; (red below 0.3, green above 0.7 and yellow in between these values). If the average reactivity is above 0.7 in the majority of repeats the average reactivity is recorded next to the position, else a null value of -999 is recorded. The efficiency of LNA annealing is evidenced by reverse transcription pausing at position 260. In the central region without SHAPE data published biochemical data from previous interrogations of the structure were used from Harrison and Lever (1992), Damgaard et al. (1998), Paillart et al. (2004) and Watts et al. (2009). The structure derived from these restraints is shown in Figure 3.

PNA Pair	Expt	Raw data peak A				Raw Data peak B				Weight peak A	Weight peak B	Weighted peak A						Weighted peak B					
		Area	Apparent FRET efficiency	Width	Height	Area	Apparent FRET efficiency	Width	Height			Weight area	Rel. Area	Weighted FRET efficiency	Width	Height	Area :height	Weight area	Rel. area	Weighted FRET efficiency	Width	Height	Area:height
A1+D6	b	40.589	0.229	0.159	203.269	66.032	0.406	0.187	281.831	4.072	4.072	165.269	0.381	0.934	0.649	827.665	0.200	268.865	0.619	1.654	0.761	1147.548	0.234
	c	3.979	0.299	0.102	31.161	21.383	0.463	0.170	100.653	4.324	4.324	17.203	0.157	1.292	0.440	134.732	0.128	92.453	0.843	2.003	0.733	435.197	0.212
	e	19.374	0.187	0.099	156.572	85.490	0.275	0.162	422.335	1.630	1.630	31.575	0.185	0.304	0.161	255.176	0.124	139.329	0.815	0.449	0.263	688.310	0.202
	el	2.405	0.316	0.080	23.866	14.663	0.460	0.158	73.908	3.214	3.214	7.731	0.141	1.015	0.258	76.710	0.101	47.130	0.859	1.479	0.509	237.554	0.198
	f	30.519	0.179	0.183	132.864	100.92	0.424	0.267	301.269	4.529	4.529	138.220	0.232	0.810	0.830	601.740	0.230	457.091	0.768	1.918	1.211	1364.449	0.335
	gl	91.106	0.201	0.181	401.332	318.097	0.422	0.253	1004.824	4.326	4.326	394.089	0.223	0.869	0.783	1736.009	0.227	1375.965	0.777	1.826	1.093	4346.486	0.317
	g	37.817	0.180	0.189	159.682	232.585	0.420	0.298	622.532	4.518	4.518	170.859	0.140	0.812	0.854	721.450	0.237	1050.829	0.860	1.896	1.347	2812.623	0.374
	h	47.168	0.190	0.161	233.690	208.99	0.361	0.302	552.329	3.285	3.285	154.963	0.184	0.624	0.529	767.755	0.202	686.624	0.816	1.187	0.992	1814.594	0.378
	hl	37.354	0.280	0.303	98.292	1.986	0.125	0.034	0.034	4.497		167.995	1.000	1.257	1.364	442.053	0.380						
	j	28.475	0.166	0.220	103.217	112.20	0.363	0.286	313.298	3.746	3.746	106.667	0.202	0.623	0.825	386.642	0.276	420.318	0.798	1.359	1.070	1173.591	0.358
		Mean	0.223			Mean	0.372		Total	38.141	33.643		Total	8.540		Total	2.103	4538.603	Total	13.771	7.978	14020.35	2.609
													AW	0.224		AW	0.055	134.903	AW	**0.409**	AW	416.734	**0.078**
	a	1.225	0.163	0.163	6.008	31.509	0.506	0.307	81.949	3.399	3.399	4.163	0.037	0.552	0.553	20.419	0.204	107.099	0.963	1.719	1.043	278.541	0.384
A1+D2	b	210.806	0.389	0.232	724.350	19.536	0.551	0.092	169.935	3.338	3.338	703.710	0.915	1.297	0.775	2418.021	0.291	65.216	0.085	1.838	0.306	567.277	0.115
	d	18.740	0.326	0.122	123.000	16.730	0.420	0.131	101.548	3.116	3.116	58.396	0.528	1.017	0.379	383.280	0.152	52.133	0.472	1.308	0.410	316.432	0.165
	dl	103.097	0.301	0.144	571.641	11.050	0.443	0.146	60.206	3.179	3.179	327.703	0.903	0.957	0.457	1817.023	0.180	35.123	0.097	1.408	0.465	191.373	0.184
	e	10.898	0.465	0.255	34.118	3.473	0.465	0.012	234.345		3.548								1.000	1.650	0.042	831.388	0.015
	g	6.759	0.374	0.012	458.969	52.783	0.356	0.190	221.503	3.173		21.447	1.000	1.186	0.037	1456.474	0.015						
	gl	70.106	0.283	0.133	420.575	54.288	0.366	0.171	253.282	3.113	3.113	218.244	0.564	0.880	0.414	1309.272	0.167	169.002	0.436	1.139	0.532	788.481	0.214
			0.299				0.427			19.318	19.693	1333.663		5.889	2.616	7404.488	1.009	440.893		9.062	2.798	2973.492	1.077
											AW	34.967		**0.305**		194.136	**0.052**	13.105		0.460		88.383	0.055
	a	18.107	0.177	0.118	122.677	77.407	0.452	0.217	284.797	4.651	4.651	84.221	0.190	0.825	0.548	570.602	0.148	360.039	0.810	2.101	1.009	1324.664	0.272
A1+D3	b	13.439	0.296	0.113	95.061	101.309	0.424	0.188	430.042	3.636	3.636	48.860	0.117	1.075	0.410	345.606	0.141	368.321	0.883	1.543	0.683	1563.468	0.236
	c	17.352	0.224	0.138	99.970	191.538	0.483	0.232	658.857	4.220	4.220	73.231	0.083	0.946	0.584	421.902	0.174	808.343	0.917	2.037	0.979	2780.551	0.291
	cl	31.401	0.251	0.149	167.669	221.612	0.477	0.214	824.499	4.142	4.142	130.050	0.124	1.041	0.619	694.415	0.187	917.825	0.876	1.977	0.888	3414.729	0.269
	d	83.771	0.466	0.217	308.708	6.780	0.467	0.042	127.478		4.267							28.931	1.000	1.991	0.181	543.975	0.053
	e	145.883	0.436	0.209	556.201	51.000	0.477	0.102	397.165		3.682							187.783	1.000	1.755	0.377	1462.361	0.128
	el	87.048	0.446	0.185	376.292	24.742	0.485	0.066	299.304		3.146							77.828	1.000	1.525	0.207	941.491	0.083
	f	86.785	0.484	0.129	537.268	8.440	0.476	0.030	228.094		3.354							28.310	1.000	1.598	0.099	765.064	0.037
	g	15.727	0.517	0.164	76.557	11.809	0.712	0.158	59.544	4.732	4.732	74.428	0.571	2.445	0.776	362.306	0.205	55.888	0.429	3.368	0.749	281.792	0.198
			0.293				0.495			21.381	35.830	410.791		6.333	2.937	2394.832	0.855	2833.269		17.895	5.173	13078.09	1.566
												10.770		0.296		62.789	0.040	84.215		**0.499**		388.727	**0.044**
A5+D6	a	87.015	0.406	0.182	381.712	10.488	0.476	0.055	153.000	3.593	3.593	312.683	0.892	1.457	0.654	1371.662	0.228	37.690	0.108	1.712	0.197	549.798	0.069
	al	12.691	0.330	0.114	88.458	32.542	0.466	0.129	201.356	3.583	3.583	45.474	0.281	1.181	0.410	316.963	0.143	116.602	0.719	1.670	0.462	721.496	0.162
	b	4.995	0.336	0.130	30.656	31.467	0.478	0.150	167.156	3.579	3.579	17.879	0.137	1.203	0.465	109.730	0.163	112.632	0.863	1.712	0.538	598.317	0.188
	d	1.170	0.434	0.041	23.019	28.039	0.503	0.229	97.691	3.750	3.750	4.387	0.040	1.629	0.152	86.326	0.051	105.153	0.960	1.885	0.859	366.366	0.287
	dl	27.962	0.503	0.218	102.464	2.389	0.476	0.041	46.720		3.757							8.973	1.000	1.788	0.153	175.505	0.051
	f	22.790	0.474	0.170	107.014	3.220	0.476	0.040	64.770		3.946							12.707	1.000	1.877	0.157	255.586	0.050
	fl	40.808	0.479	0.204	159.573	2.812	0.483	0.038	59.744		4.439							12.480	1.000	2.143	0.167	265.176	0.047
	g	39.885	0.473	0.216	147.337	13.941	0.465	0.012	909.921		3.635							50.675	1.000	1.689	0.044	3307.436	0.015
	gl	96.786	0.451	0.249	310.047	17.903	0.466	0.012	1237.18		3.973							71.131	1.000	1.853	0.046	4915.517	0.014
	h	2.088	0.228	0.063	26.637	21.818	0.384	0.207	83.976	3.538	3.538	7.387	0.087	0.806	0.221	94.239	0.078	77.189	0.913	1.359	0.733	297.096	0.260
	i	15.028	0.266	0.143	83.717	142.85	0.489	0.214	532.588	5.111	5.111	76.814	0.095	1.358	0.732	427.900	0.180	730.143	0.905	2.498	1.094	2722.201	0.268
			0.398				0.469			23.155	42.905	464.625		7.634	2.635	2406.818	0.843	1335.375		20.185	4.449	14174.49	1.411
												12.182		0.330		63.104	0.036	39.692		**0.470**		421.316	**0.033**

PNA Pair	Expt	Raw data peak A				Raw data peak B				Weight peak A	Weight peak B	Weighted peak A						Weighted peak B					
		Area	Apparent FRET efficiency	Width	Height	Area	Apparent FRET efficiency	Width	Height			Weighted area	Rel. area	Weighted FRET efficiency	Width	Height	Area :height	Weighted area	Rel. area	Weighted FRET efficiency	Width	Height	Area:height
A5+D2	b	20.587	0.496	0.236	69.590	6.892	0.466	0.012	467.436		4.384							30.215	1.000	2.044	0.052	2049.434	0.015
	c	1.725	0.119	0.143	9.660	21.988	0.301	0.239	73.472	3.269	3.269	5.641	0.073	0.390	0.466	31.580	0.179	71.881	0.927	0.985	0.781	240.192	0.299
	d	0.958	0.328	0.104	7.324	4.453	0.474	0.135	26.221	3.249	3.249	3.111	0.177	1.066	0.339	23.795	0.131	14.468	0.823	1.541	0.440	85.192	0.170
	dl	75.355	0.404	0.247	243.567	6.844	0.451	0.105	52.078	3.222	3.222	242.764	0.917	1.303	0.795	784.672	0.309	22.050	0.083	1.453	0.338	167.774	0.131
	f	44.206	0.368	0.173	203.772	1.618	0.457	0.043	30.129	3.093	3.093	136.723	0.965	1.138	0.535	630.241	0.217	5.005	0.035	1.413	0.133	93.186	0.054
	h	5.021	0.259	0.125	32.071	31.759	0.448	0.213	119.037	3.609	3.609	18.122	0.137	0.936	0.451	115.754	0.157	114.628	0.863	1.618	0.768	429.637	0.267
	hl	12.002	0.239	0.167	57.222	71.557	0.450	0.240	237.692	3.479	3.479	41.760	0.144	0.831	0.582	199.101	0.210	248.979	0.856	1.566	0.836	827.041	0.301
	l	2.971	0.237	0.135	17.567	15.904	0.439	0.175	72.335	3.448	3.448	10.243	0.157	0.819	0.465	60.564	0.169	54.831	0.843	1.515	0.605	249.380	0.220
	il	20.611	0.391	0.210	78.396	2.626	0.493	0.066	31.843	3.364	3.364	69.344	0.887	1.315	0.706	263.751	0.263	8.834	0.113	1.659	0.221	107.131	0.082
			0.316				0.442			26.733	31.118	527.709		7.797	4.340	2109.459	1.634	570.891		13.795	4.173	4248.967	1.539
												13.836		0.292		55.307	0.061	16.969		**0.443**		126.294	**0.049**
	a	92.108	0.467	0.205	357.905	6.336	0.487	0.055	92.034		4.639							29.394	1.000	2.259	0.255	426.963	0.069
A5+D3	b	2.746	0.243	0.091	24.040	33.391	0.480	0.227	117.436	3.925	3.925	10.779	0.076	0.953	0.358	94.363	0.114	131.066	0.924	1.885	0.890	460.958	0.284
	c	59.416	0.373	0.205	231.585	17.172	0.460	0.087	156.650	3.210	3.210	190.735	0.776	1.198	0.657	743.424	0.257	55.124	0.224	1.476	0.281	502.870	0.110
	cl	13.913	0.465	0.012	943.172	64.319	0.449	0.342	150.269		4.015							258.217	1.000	1.803	1.371	603.277	0.428
	d	51.270	0.449	0.157	261.075	5.720	0.470	0.052	87.458		3.664							20.959	1.000	1.722	0.191	320.448	0.065
	e	19.143	0.214	0.170	89.976	37.297	0.448	0.194	153.743	3.085	3.085	59.056	0.339	0.659	0.524	277.578	0.213	115.062	0.661	1.383	0.597	474.300	0.243
	f	37.546	0.356	0.208	144.094	9.074	0.468	0.126	57.483	3.289	3.289	123.470	0.805	1.169	0.684	473.857	0.261	29.838	0.195	1.540	0.414	189.034	0.158
	h	1.895	0.482	0.121	12.463	13.322	0.545	0.261	40.705	4.440	4.440	8.415	0.125	2.140	0.539	55.335	0.152	59.147	0.875	2.420	1.159	180.728	0.327
			0.381				0.476			17.949	30.267	392.455		6.119	2.761	1644.556	0.996	698.808		14.490	5.159	3158.579	1.684
												10.290		0.341		43.118	0.056	20.771		**0.479**		93.884	**0.056**
	a	5.162	0.345	0.012	353.020	20.206	0.483	0.304	53.049	4.765	4.765	24.599	0.203	1.642	0.056	1682.215	0.015	96.288	0.797	2.300	1.448	252.788	0.381
A4+D6	b	7.205	0.168	0.210	27.339	42.653	0.455	0.289	117.750	3.961	3.961	28.537	0.145	0.665	0.833	108.289	0.264	168.951	0.855	1.804	1.145	466.410	0.362
	bl	5.668	0.180	0.180	25.101	58.429	0.453	0.328	142.154	3.702	3.702	20.981	0.088	0.665	0.667	92.914	0.226	216.277	0.912	1.675	1.214	526.193	0.411
	d	98.297	0.402	0.190	413.406	1.186	0.626	0.122	7.752	3.379	3.379	332.125	0.988	1.357	0.641	1396.815	0.238	4.007	0.012	2.113	0.412	26.193	0.153
	dl	15.932	0.436	0.096	132.963	68.506	0.410	0.187	291.581		3.283							224.904	1.000	1.347	0.615	957.255	0.235
	e	31.548	0.290	0.137	184.169	19.859	0.424	0.194	81.868	3.810	3.810	120.194	0.614	1.107	0.521	701.660	0.171	75.659	0.386	1.617	0.737	311.907	0.243
	el	64.838	0.274	0.136	381.073	39.309	0.392	0.195	160.454	3.510	3.510	227.579	0.623	0.963	0.477	1337.553	0.170	137.974	0.377	1.374	0.686	563.190	0.245
			0.299				0.463			23.126	26.409	754.015		6.398	3.194	5319.445	1.083	924.062		12.230	6.258	3103.937	2.030
												19.769		0.277		139.469	0.047	27.466		**0.463**		92.260	**0.077**
A4+D2	c	92.123	0.355	0.223	329.369	2.260	0.473	0.040	45.128	3.823	3.823	352.187	0.976	1.359	0.853	1259.180	0.280	8.641	0.024	1.809	0.153	172.525	0.050
	d	103.339	0.339	0.251	328.368	71.821	0.438	0.150	381.122	3.855	3.855	398.356	0.590	1.307	0.968	1265.813	0.315	276.860	0.410	1.690	0.580	1469.171	0.188
	e	39.640	0.242	0.152	207.685	72.116	0.378	0.239	240.753	3.606	3.606	142.924	0.355	0.874	0.549	748.811	0.191	260.014	0.645	1.362	0.862	868.041	0.300
	el	113.569	0.383	0.190	382.327					3.528	3.528	400.678	1.000	1.352	0.671	1348.875	0.297						
	f	132.306	0.395	0.218	483.871	5.799	0.542	0.077	60.154	3.721	3.721	492.304	0.958	1.470	0.812	1800.463	0.273	21.579	0.042	2.017	0.286	223.829	0.096
	fl	104.076	0.409	0.215	385.665	4.978	0.459	0.036	110.555	4.188	4.188	435.870	0.954	1.713	0.902	1615.164	0.270	20.850	0.046	1.924	0.150	463.006	0.045
	g	4.622	0.466	0.012	310.699	7.103	0.551	0.235	24.111	2.833	2.833	13.092	0.394	1.321	0.034	880.071	0.015	20.119	0.606	1.561	0.666	68.294	0.295
	h	6.601	0.471	0.141	37.457	8.747	0.626	0.207	33.772	3.381	3.381	22.318	0.430	1.594	0.475	126.631	0.176	29.570	0.570	2.115	0.699	114.172	0.259
	j	2.479	0.492	0.050	39.680	24.184	0.542	0.254	75.867	6.097	6.097	15.115	0.093	3.002	0.304	241.944	0.062	147.456	0.907	3.306	1.551	462.586	0.319
	k	38.492	0.481	0.211	145.270	13.843	0.650	0.175	63.221	5.623	5.623	216.439	0.735	2.706	1.189	816.855	0.265	77.840	0.265	3.657	0.982	355.491	0.219
			0.404				0.518			40.654	40.654	2489.283		16.698	6.756	10103.80	2.144	862.929		19.440	5.928	4197.115	1.771
												65.266		**0.411**		264.909	**0.053**	25.649		0.478		124.753	0.044

Table S2. Calculation of weighted efficiencies from signal:noise ratio.
Yellow highlighting indicates where the peak was deemed insignificantly small or at the same center position and so was removed. A is the left peak, B is the right peak. AW = after weighting. Area:height ratio was calculated for each weighted repeat and used as flexibility information to more accurately model the structure (highlighted in bold). The mean weighted FRET efficiency was used to calculate the monomer distance between the pair. The decision as to which peak represented the monomeric species was made based upon the shift in peak relative area on the addition of DIS LNA (Figure S3). The final efficiencies, distances and tolerances are shown in Table 1.

Pair	Random starting models			FRET distance constrained			Minimized		Energy	Relaxed		Energy
	Distance	Diff.	$\bar{x}$	Distance	Diff.	$\bar{x}$	Distance	Diff.	$\bar{x}$ distance	Distance	Diff.	$\bar{x}$ distance
	r0											
A1+D2	36.069	52.618		83.568	5.119		83.036	5.651	1466.864	87.02	1.667	1229.800
A1+D3	11.153	54.239		63.486	1.906		62.960	2.432		58.04	7.352	
A1+D6	93.991	22.980		67.324	3.687		66.576	4.435		59.64	11.371	
A5+D2	24.088	46.888		73.378	2.402		74.278	3.302		89.91	18.934	
A5+D3	57.319	12.661		70.784	0.804		71.588	1.608		63.54	6.440	
A5+D6	56.038	6.399		63.876	1.439		63.453	1.016		57.24	5.197	
A4+D2	45.723	27.613		68.800	4.536		68.589	4.747		78.29	4.954	
A4+D6	98.376	28.733	31.516	77.156	7.513	3.426	76.918	7.275	3.808	81.52	11.877	8.474
	r1											
A1+D2	75.340	13.347		84.420	4.267		84.643	4.044	1376.147	91.41	2.723	1159.738
A1+D3	37.394	27.998		67.301	1.909		67.252	1.860		64.96	0.432	
A1+D6	25.946	45.065		70.542	0.469		71.081	0.070		67.57	3.441	
A5+D2	84.496	13.520		73.381	2.405		74.276	3.300		86.85	15.874	
A5+D3	46.532	23.448		67.393	2.587		67.302	2.678		67.13	2.850	
A5+D6	19.025	43.412		63.892	1.455		64.468	2.031		67.38	4.943	
A4+D2	37.964	35.372		67.941	5.395		67.769	5.567		67.12	6.216	
A4+D6	62.345	7.298	26.182	69.484	0.159	2,331	69.401	0.242	2.474	64.98	4.663	5.143
	r2											
A1+D2	72.688	15.999		84.995	3.692		84.624	4.063	1376.687	85.48	3.207	1200.134
A1+D3	32.141	33.251		63.478	1.914		63.228	2.164		59.85	5.542	
A1+D6	62.520	8.491		74.700	3.689		74.914	3.903		91.57	20.559	
A5+D2	63.990	6.986		73.349	2.373		73.348	2.372		82.32	11.344	
A5+D3	17.415	52.565		71.377	1.397		70.938	0.958		65.13	4.850	
A5+D6	82.780	20.343		63.875	1.438		63.528	1.091		74.44	12.003	
A4+D2	65.722	7.614		70.308	3.028		70.137	3.199		70.13	3.206	
A4+D6	87.383	17.740	20.374	77.163	7.520	3.131	76.928	7.285	3.129	89.09	19.447	10.020
	r3											
A1+D2	62.486	26.201		93.835	5.148		93.796	5.109	1332.717	95.940	7.253	1179.79
A1+D3	24.440	40.952		63.421	1.971		62.739	2.653		58.057	7.335	
A1+D6	81.940	10.929		67.364	3.647		66.856	4.155		64.414	6.597	
A5+D2	15.008	55.968		68.606	2.370		68.448	2.528		70.637	0.339	
A5+D3	27.844	42.136		67.930	2.050		67.665	2.315		59.829	10.151	
A5+D6	95.033	32.596		60.974	1.463		60.921	1.516		60.936	1.501	
A4+D2	38.049	35.287		68.070	5.266		67.889	5.447		69.122	4.214	
A4+D6	81.187	11.544	31.952	77.202	7.559	3.684	77.249	7.606	3.916	92.633	22.990	7.547
	r4											
A1+D2	86.923	1.764		93.796	5.109		93.957	5.270	1507.232	95.09	6.403	1235.324
A1+D3	41.038	24.354		63.461	1.931		62.869	2.523		58.03	7.362	
A1+D6	79.941	8.930		67.371	3.640		66.987	4.024		71.18	0.169	
A5+D2	86.178	15.202		73.365	2.389		74.124	3.148		71.38	0.404	
A5+D3	15.063	54.917		67.359	2.621		66.801	3.179		53.28	16.700	
A5+D6	35.243	27.194		63.916	1.479		63.364	0.927		70.55	8.113	
A4+D2	81.946	8.610		70.434	2.902		70.616	2.720		71.71	1.626	
A4+D6	45.328	24.315	20.661	77.199	7.556	3.453	77.019	7.376	3.646	87.54	17.897	7.334
	r5											
A1+D2	12.587	76.100		30.554	58.133		30.218	58.469	1436.520	31.337	57.350	1184.628
A1+D3	66.966	1.574		67.305	1.913		66.031	0.639		55.785	9.607	
A1+D6	75.049	4.038		67.285	3.726		66.584	4.427		57.881	13.130	
A5+D2	69.011	1.965		73.386	2.410		73.880	2.904		103.812	32.836	
A5+D3	22.007	47.973		72.557	2.577		72.741	2.761		89.495	19.515	
A5+D6	32.053	30.384		63.893	1.456		63.803	1.366		77.828	15.391	
A4+D2	77.908	4.572		67.196	6.140		66.667	6.669		59.126	14.210	
A4+D6	47.086	22.557	23.645	69.489	0.154	9.564	69.331	0.312	9.693	63.695	5.948	20.998
	r6											
A1+D2	51.985	36.702		83.581	5.106		83.361	5.326	1381.984	94.309	5.622	1182.643
A1+D3	46.080	19.312		63.486	1.906		62.708	2.684		62.311	3.081	
A1+D6	45.087	25.924		67.337	3.674		66.795	4.216		68.903	2.108	
A5+D2	34.012	36.964		73.376	2.400		73.368	2.392		80.234	9.258	
A5+D3	57.874	12.106		67.361	2.619		67.231	2.749		43.222	26.758	
A5+D6	80.966	18.529		63.924	1.487		64.131	1.694		64.954	2.517	
A4+D2	38.391	34.945		72.079	1.257		72.531	0.805		76.818	3.482	
A4+D6	81.594	11.951	24.544	77.180	7.537	3.248	77.518	7.875	3.468	83.642	13.999	8.353

Pair	Random starting models			FRET distance constrained			Minimized		Energy	Relaxed		Energy
	Distance	Diff.	$\bar{x}$	Distance	Diff.	$\bar{x}$	Distance	Diff.	$\bar{x}$ distance	Distance	Diff.	$\bar{x}$ distance
	r7											
A1+D2	7.339	81.348		83.553	5.134		82.961	5.726	1472.871	80.928	7.759	1179.881
A1+D3	28.482	36.910		63.486	1.906		62.292	3.100		55.633	9.759	
A1+D6	70.992	0.019		67.296	3.715		66.416	4.595		52.403	18.608	
A5+D2	65.430	5.546		73.364	2.388		74.403	3.427		91.768	20.792	
A5+D3	56.903	13.077		67.410	2.570		68.249	1.731		70.398	0.418	
A5+D6	59.029	3.408		63.889	1.452		63.761	1.324		70.847	8.410	
A4+D2	24.010	49.326		69.186	4.150		69.586	3.750		68.487	4.849	
A4+D6	82.560	12.917	25.319	70.476	0.833	2.768	70.549	0.906	3.070	74.660	5.017	9.451
	r8											
A1+D2	53.017	35.670		92.805	4.118		92.153	3.466	1436.724	94.268	5.581	1190.774
A1+D3	79.874	14.482		63.473	1.919		63.159	2.233		55.272	10.120	
A1+D6	73.412	2.401		73.534	2.523		73.559	2.548		75.982	4.971	
A5+D2	66.905	4.071		73.370	2.394		74.540	3.564		90.480	19.504	
A5+D3	57.017	12.963		67.374	2.606		67.436	2.544		63.304	6.676	
A5+D6	89.885	27.448		60.992	1.445		60.757	1.680		57.946	4.491	
A4+D2	39.009	34.327		72.082	1.254		72.450	0.886		77.854	4.518	
A4+D6	135.890	66.247	24.701	77.182	7.539	2.975	77.298	7.655	3.072	84.129	14.486	8.793
	r9											
A1+D2	93.858	5.171		83.549	5.138		83.277	5.410	1453.055	80.921	7.766	1195.877
A1+D3	87.284	21.892		64.120	1.272		63.746	1.646		57.948	7.444	
A1+D6	29.935	41.076		67.334	3.677		67.151	3.860		70.721	0.290	
A5+D2	64.704	6.272		73.420	2.444		73.780	2.804		97.238	26.262	
A5+D3	33.338	36.642		71.821	1.841		71.601	1.621		85.812	15.832	
A5+D6	39.311	23.126		63.910	1.473		63.511	1.074		71.813	9.376	
A4+D2	66.753	6.583		69.295	4.041		69.135	4.201		65.478	7.858	
A4+D6	68.857	0.786	17.693	77.188	7.545	3.429	77.739	8.096	3.589	86.911	17.268	11.512

Table S3. Table of the difference between the modelled and measured distances for each fluorophore pair from starting models 0-9.

The ‘Random starting models’ columns show the pseudo experimental FRET distances and how much they differ from experimental ones (Diff). The ‘FRET distance constrained’ columns are after simulated annealing constrained by the FRET measured distances. The ‘Minimized’ columns are from a minimization step which ensures the correct shape of helices. The Relaxed columns represent a rigorous room temperature anneal without FRET constraints.

Due to the added complexity of modeling the PNA, the glycol linker and the fluorophore with the RNA, the distances used for modeling were between the C1’atoms of the nucleotide closest to the fluorophore. A description of randomized starting models is shown in Figure S4 and shown in Figure 4. See also Tables 3 and 4.

Supplemental Experimental Procedures

Anisotropy Measurements

Steady-state fluorescence spectra were measured on a Cary Eclipse Fluorometer, Varian Inc. The excitation and emission band-passes were set to 5 nm. 150 ml samples of labeled PNA on HIV RNA at 20 nM concentration were excited and emission spectra were collected. All spectra were corrected for instrument response. Fluorescence anisotropies for donor (Atto488) and acceptor (Atto647) were calculated from the polarization of the emission components I_{VV}, I_{VH}, I_{HV}, and I_{HH} (where the subscripts denote the orientation of the excitation and emission polarizers) as $r = (I_{VV} - GI_{VH})/(I_{VV} + 2GI_{VH})$, where $G = I_{HV}/I_{HH}$. Where *IVV* is fluorescence intensity with excitation polarization aligned vertically and emission excitation polarization aligned vertically, *IVH* is fluorescence intensity recorded with the emission polarization aligned horizontally. For Atto488 anisotropy, excitation was at 480 nm and emission spectra were collected at 525 nm; whereas for Atto647 anisotropy, the excitation wavelength was set to 635 nm and emission was collected at 670 nm. All measurements were performed at 21°C. The Förster distance of donor and acceptor pair is normally calculated using an orientation factor κ^2 with an average value of 2/3 for free rotating dyes. This is not appropriate for these experiments as neither dye has free rotation, most probably due to both the linker joining the dye to the PNA and due to some attraction between the dye and RNA. Atto647 had the least rotational freedom of the dyes used and so it was given a 0° dipole orientation and the relative donor angle (Atto647) was calculated.

We then recalculated the Förster distance for this particular structure based on the emission anisotropy from the FRET measurements, which is a product of the limiting anisotropy (Figure S2), the donor and acceptor axial depolarization factors d^x_d and d^x_a , and the depolarization factor, $P_2(\cos \beta_{da})$, corresponding to the angle between the donor and acceptor symmetry axes (Ivanov and Mizuuchi, 2009). The emission anisotrpy between fluorophores is EA, the angle from the donor-acceptor vector is θ, the orientation factor is κ^2.

$$EA_{FRET} = \tfrac{2}{5} d^x_d d^x_a P_2 \cos(\beta_{da}) \qquad \textbf{Equation 1}$$

So we get,

$$\cos(\beta_{da}) = \sqrt{\tfrac{1}{3} + \tfrac{5}{3}\left(\frac{EA_{FRET}}{d^x_d d^x_a}\right)} \qquad \textbf{Equation 2}$$

where,

$$d^x_d = \sqrt{\tfrac{5}{2} r_d} \text{ and } d^x_a = \sqrt{\tfrac{5}{2} r_a} \qquad \textbf{Equation 3}$$

with the angle β_{da} The orientation factor κ^2 of this dye pair can be recalculated by:

$$k^2 = (3\cos \vartheta_a \vartheta_d - \cos \beta_{da})^2 \qquad \textbf{Equation 4}$$

The Δ factor to correct the Förster distance was calculated by:

$$\Delta \equiv \sqrt[6]{k^2 / (2/3)} \qquad \textbf{Equation 5}$$

The K^2 range was calculated by:

$$k^2_{min} = 2/3\left(1 - \frac{\sqrt{\frac{5}{2}EA_d} + \sqrt{\frac{5}{2}EA_a}}{2}\right) \qquad \textbf{Equation 6}$$

$$k^2_{max} = 2/3\left(1 + \sqrt{\frac{5}{2}EA_d} + \sqrt{\frac{5}{2}EA_a} + 3\sqrt{\frac{5}{2}EA_d}\sqrt{\frac{5}{2}EA_d}\right) \qquad \textbf{Equation 7}$$

Using the equations 6 and 7, we calculated $kmin^2$ =0.172 and $kmax^2$ =2.088.

Shot Noise

The random fluctuations in fluorescence signal intensity in both channels due to photon shot-noise results in uncertainty in the true value of the Apparent FRET efficiencies, $\boldsymbol{E}_{\mathbf{app.}}$ The values can be estimated using a standard analysis of error. The width ΔR from shot-noise contribution to the $E(_{app})$ histogram was given by the following formula (Dahan et al., 1999, Ying et al., 2000):

Where $$\Delta R = \left(\frac{\gamma(1-\gamma)^2}{T} + \frac{\gamma^2(1-\gamma)}{T} \right)^{1/2}$$

$\gamma = \overline{Ia}/(\overline{Ia} + \overline{Id})$, and T is the threshold value per ms in all experiments. The upper limit for the shot-noise width ΔR was estimated to be 0.12 for a 40 base model dsDNA sample. Experimental width from the model ds-DNA sample is approximately twice the magnitude of the shot-noise induced width. The histogram width of HIV-RNA FRET samples in Table S2 is often wider than the width of the model ds-DNA. These results indicate that there is some other fluctuation process contributing to the $E(_{app})$ histogram. As the linker is identical in all cases, we suggest that the flexibility of the RNA linking the two dyes in the monomer may be the second reason that makes the histogram wider based on this experimental and the theoretical study.

Supplemental References

Dahan, M., Deniz, A.A., Ha, T.J., Chemla, D.S., Schultz, P.G., and Weiss, S. (1999). Ratiometric measurement and identification of single diffusing molecules. Chem. Phys. 247, 85–106.

Hofacker, I.L., Fontana, W., Stadler, P.F., Bonhoeffer, L.S., Tacker, M., and Schuster, P. (1994). Fast folding and comparison of RNA secondary structures. Chemical Monthly 125, 167–188.

Ivanov, V., Li, M., and Mizuuchi, K. (2009). Impact of emission anisotropy on fluorescence spectroscopy and FRET distance measurements. Biophys. J. 97, 922–929.

Smith, C., Heyne, S., Richter, A.S., Will, S., and Backofen, R. (2010). Freiburg RNA tools: a web server integrating IntaRNA, ExpaRNA and LocRNA. Nucleic Acids Res. 38, 373–377.

Ying, L.M., Wallace, M.I., Balasubramanian, S., and Klenerman, D. (2000). Ratiometric analysis of single-molecule fluorescence resonance energy transfer using logical combinations of threshold criteria: A study of 12-mer DNA. J. Phys. Chem. B 104, 5171–5178.
